# Integrating the Reverse Boudouard Reaction for a More Efficient Green Methanol Synthesis from CO_2_ and Renewable Energy

**DOI:** 10.1021/acssuschemeng.5c01021

**Published:** 2025-05-08

**Authors:** Juan D. Medrano-García, Marina T. Chagas, Gonzalo Guillén-Gosálbez

**Affiliations:** † Institute for Chemical and Bioengineering, Department of Chemistry and Applied Biosciences, ETH Zurich, Vladimir Prelog Weg 1, Zurich 8093, Switzerland; †† NCCR Catalysis, Zurich 8093, Switzerland

**Keywords:** CO_2_ hydrogenation, reverse Boudouard reaction (RB), biochar, life cycle assessment (LCA), climate change, process simulation, win-win scenario, process integration

## Abstract

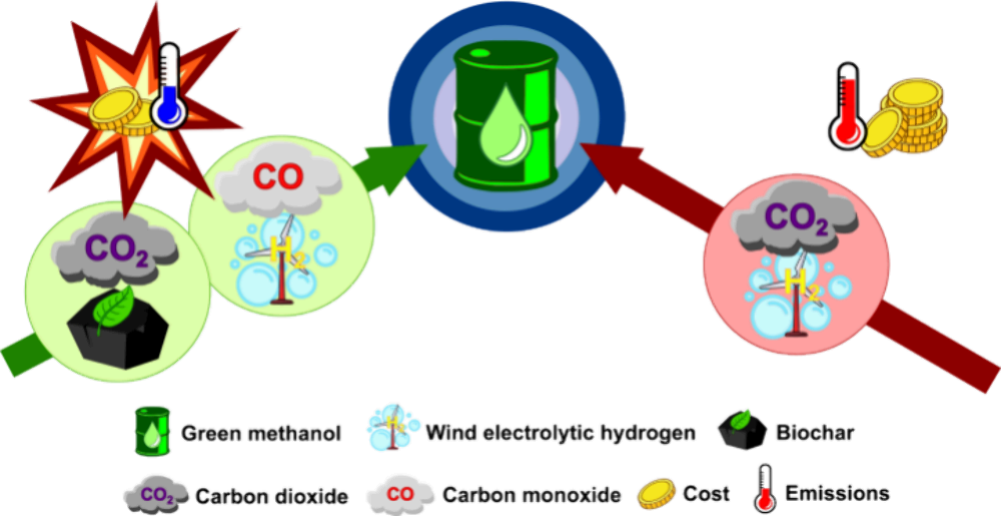

Green methanol is an important renewable platform chemical that could be used to produce a wide range of sustainable products and fuels. However, it is currently economically unappealing. This high cost is mainly driven by the CO_2_ hydrogenation process, which requires 50% more H_2_ consumption than the classic fossil-based CO-rich syngas to methanol. To overcome this limitation, here we evaluate the economic and environmental implications of producing green methanol from electrolytic H_2_ and captured CO_2_ integrated with the reverse Boudouard (RB) reaction. We designed an integrated process based on a standard green methanol plant, adding an RB reactor to reduce CO_2_ to CO using biochar prior to the methanol synthesis loop. Combining process simulation with life cycle assessment, we find that integrating both technologies leads to an economic and environmental win-win scenario compared with the base green methanol case. More specifically, production costs are decreased by 5% in an expanded system that assumes the simultaneous production of methanol, biogenic hydrogen, and industrial high-temperature heating under both scenarios. Furthermore, this alternative synthesis shows a reduced carbon footprint of 5% and a 4 to 10% improvement in human health, ecosystems quality, and resource scarcity, revealing no significant probability of associated burden shifting when expanding the system. Finally, when compared with fossil-based methanol, the RB integration makes green methanol competitive when H_2_ is available at 3.5–2.0 $/kg, compared to the 2.3–1.3 $/kg required for the standard green methanol configuration. Our results highlight a potentially better alternative to direct CO_2_ hydrogenation for green methanol synthesis and, in a broader context, demonstrate the benefits of integrating processes to exploit their synergies.

## Abstract 2

Integrating biochar CO_2_ gasification with green methanol synthesis results in an economic and environmental win-win due to increased material efficiency.

## Introduction

Green methanol is seen as a major player in the transition toward a more sustainable economy due to its versatility as a building block for chemicals and its potential use as a fuel.^1−4^ The synthesis of green methanol mainly relies on CO_2_ hydrogenation using electrolytic H_2_, consuming three moles of H_2_ per mole of methanol (reaction 1):
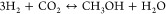
1

In contrast, the business-as-usual (BAU) fossil methanol is often produced via steam reforming (SMR) of fossil methane to yield syngas (H_2_ + CO) suitable for the methanol synthesis, with a specific H_2_ consumption (SHC) of two moles per mole of methanol (reaction 2):^5,6^
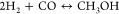
2

The main difference between both reaction pathways is the carbon source of methanol (i.e., CO_2_ for the green and CO for the fossil route), which leads to consuming up to 50% more H_2_ in the case of green methanol, making the process economically unappealing when factoring in the high cost of renewable H_2_.^7−9^

Previous works already highlighted that H_2_ constitutes the main economic and environmental hotspot in green methanol production. However, studies exploring ways to reduce such H_2_ requirements are scarce. Bampau et al.^10^ studied the effect of several process variables in a hybridized methanol process with fossil syngas and renewable H_2_ to meet different feed compositions. Among their main results, they conclude that lowering the stoichiometric syngas number (H_2_ – CO_2_)/(CO + CO_2_) of the mix below the stoichiometric value of two leads to an increased methanol production per unit of mass of H_2_ fed to the system. Medrano et al.^6^ studied the influence of an increased CO_2_ carbon fraction in fossil methane-reformed syngas fed to a methanol loop, finding that the lowest allowable fraction yields the best economic and environmental performance.

Overall, from these works, it can be extracted that the oxidation state of the carbon contained in the syngas plays a critical role in H_2_ efficiency (*i.e*., lower CO/CO_2_ ratios in the syngas lead to higher H_2_ consumption), which is consistent with reactions 1 and (2). However, none of these works delve into how to maximize this ratio when using CO_2_ and H_2_ as main feedstock.

Here, we shall explore a strategy to reduce green methanol production costs by decreasing the H_2_ requirements via the conversion of CO_2_ to CO before the methanol synthesis takes place. Several options arise to perform this task. The first alternative is found in the reverse water gas shift (RWGS) reaction, where one mole of H_2_ reduces one mole of CO_2_ into CO and water (reaction 3):
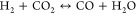
3

However, the overall H_2_ balance of reactions 2 and (3) is the same as reaction 1 as discussed by Basini et al.,^11^ who reported a SHC of around three for an integrated system of RWGS and green methanol.

Alternatively, we could deploy CO_2_ electro-reduction^12^ coupled with green methanol production to reduce the H_2_ needs. Specifically, one mole of electro-reduced CO_2_ could avoid the consumption of a mole of electrolytic H_2_ by enabling reaction 2. However, both electrolytic processes (i.e., water splitting and CO_2_ electro-reduction) share similar energy requirements based on the Gibbs free energy.^13^ Specifically, current energy efficiencies for water splitting and CO_2_ electro-reduction (ca. 80%^14^ and 60%,^15^ respectively) imply that while it would be possible to replace three moles of electrolytic H_2_ (reaction 1) with two moles of electrolytic H_2_ and one mole of CO from the electro-reduction of CO_2_ (reaction 2), such an approach would likely be unappealing. This was already discussed by Adnan and Kibria,^16^ with costs of ∼ 0.90 and ∼ 1.00 $/kg green methanol for the standard CO_2_ hydrogenation and CO_2_ hydrogenation coupled with CO_2_ electroreduction, respectively.

Alternatively, we resort here to a less conventional but well-known pathway to convert CO_2_ to CO, namely, the reverse Boudouard (RB) reaction (reaction 4):^17,18^
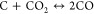
4

This reaction occurs in gasification processes involving virtually any carbon-based material and CO_2_.^19^ It is also crucial in the steel industry, where the CO_2_ produced by the combustion of coke in the blast furnace oxidizes the remaining coke to CO, which then reduces the iron oxides to metallic iron.^20^ Finally, it has also been commercialized by CAPHENIA, in a multireaction process unit where aerosol carbon produced from high-temperature methane plasma splitting reacts with CO_2_, reportedly achieving 99% conversion.^21^ However, as far as the authors are aware, a reactor that carries out exclusively this reaction has never been implemented at an industrial scale.

Our goal, hence, is to investigate the extent to which coupling the RB reaction with green methanol production could potentially decrease costs and impacts by reducing CO_2_ to CO prior to the green methanol reactor.

The (bio)char byproduct in the gasification of biomass or other carbonaceous materials is usually used for energy production, with other applications including wastewater treatment, catalysis, fuel cells and soil enhancement.^22^ Recently, it has drawn some attention as a potential carbon source in chemical synthesis. More specifically, gasification with CO_2_ via the RB reaction has been labeled as a promising option to valorize both the solid carbon and the greenhouse gas.^23^ However, the RB reaction requires high temperatures over 800 °C to displace the equilibrium toward CO formation,^19^ so the trade-off energy requirements vs H_2_ consumption needs to be carefully evaluated to properly gauge the potential of the integrated system. To this end, we carry out, for the first time, an economic and environmental study of deploying both technologies in tandem. We use process simulation to model two scenarios, i.e., a standard CO_2_ hydrogenation green methanol process (base case), and the integrated configuration with an RB reactor that considers the potential development and industrial deployment of the novel technology. Our results suggest that this integrated approach holds the potential to improve both the production costs and environmental impacts (global warming potential, human health, ecosystems quality and resource scarcity) of green methanol production leading to a win-win scenario relative to the conventional green methanol via direct CO_2_ hydrogenation.

## Methodology

We develop two process simulations in Aspen HYSYS v12 for the different green methanol scenarios. The first simulation (base case) represents the standard green methanol production from CO_2_ hydrogenation.^7^ The other methanol synthesis simulation integrates an RB reactor to convert CO_2_ into CO before reacting with the electrolytic H_2_. Additionally, we develop a third simulation in Aspen Plus v12 to model H_2_ and biochar coproduction through gasification of woody biomass. After a heat integration analysis performed with Aspen Energy Analyzer, we obtained the foreground system data (i.e., material and energy inputs of the plant) associated with green methanol and biochar production. Then, we carry out an LCA in Simapro v9.2^24^ using the background data (data from all the supply chain activities linked to the foreground) from Ecoinvent v3.5.^25^ Finally, we perform the economic assessment using the total annualized cost (TAC) of the plants.^26^ We describe first the process simulations, then the environmental assessment and finally the economic analysis.

### Green Methanol Synthesis Overview

**Figure 1:**
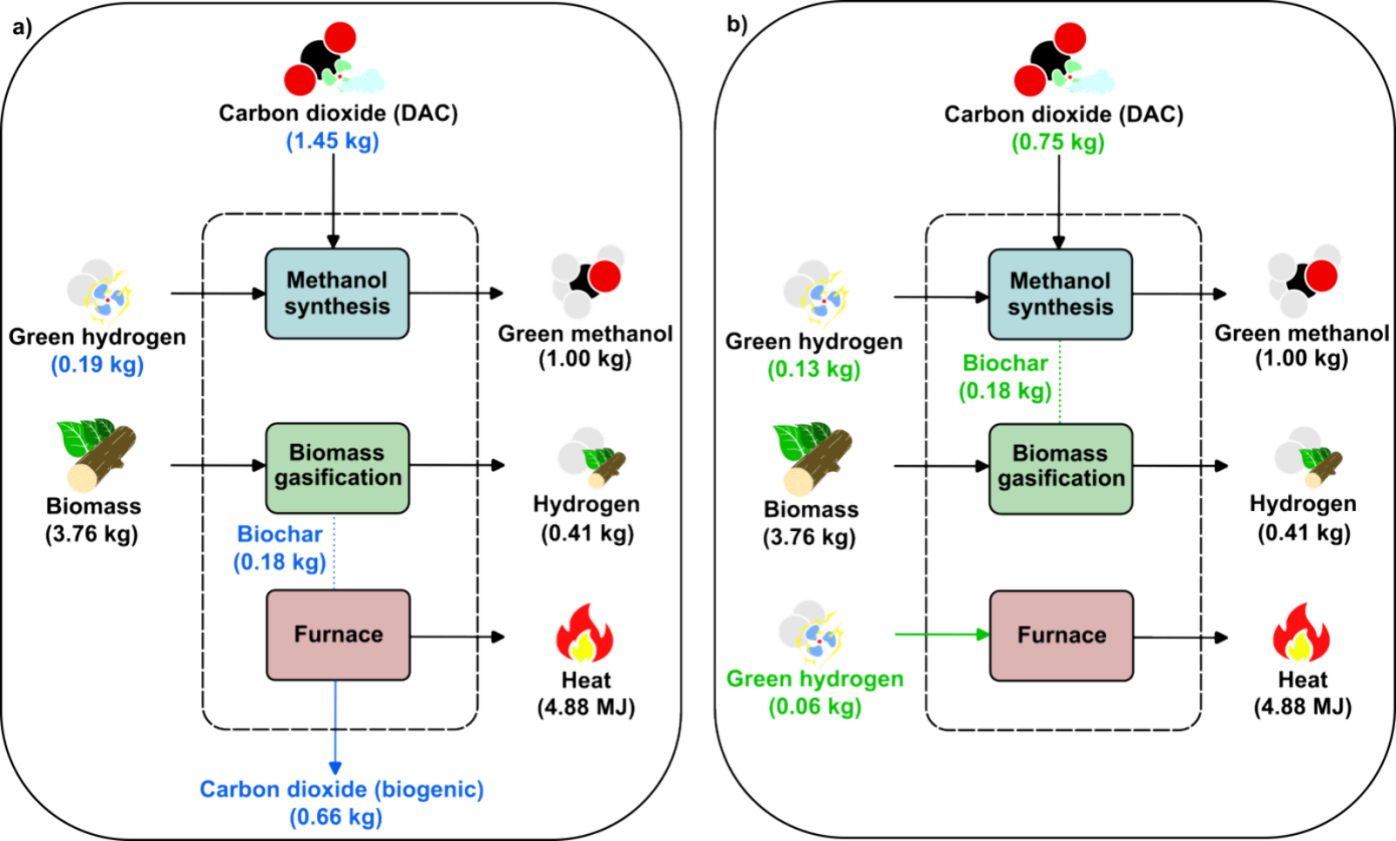
Visual representation of the system expansion of green methanol synthesis, biomass gasification to H_2_ and industrial heating scenarios: (a) base case; (b) Boudouard case. A more detailed life cycle inventory of each scenario can be found in Section B of the Supporting Information.

**Figure 2:**
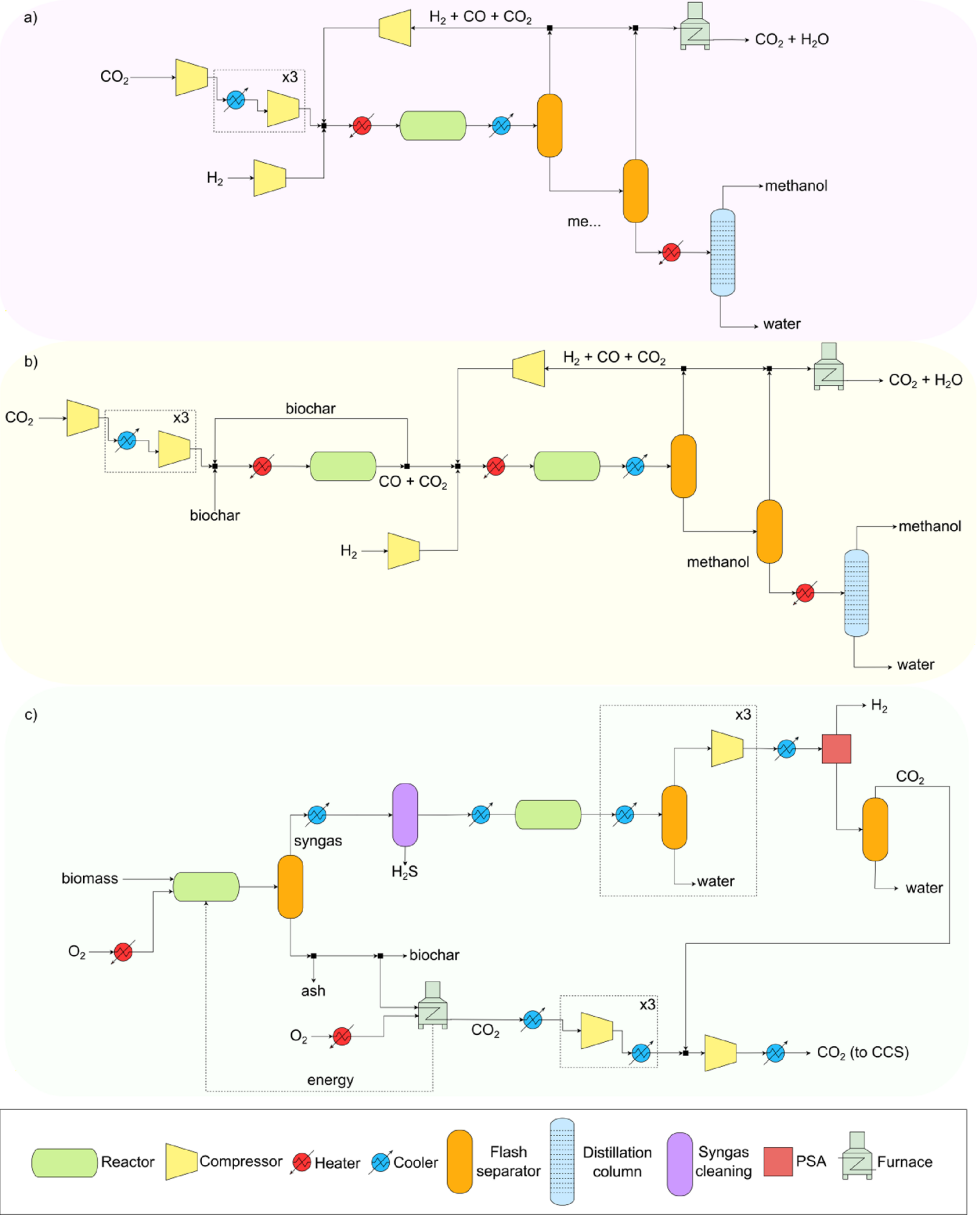
Process simulation flowsheet overview: (a) Green methanol synthesis; (b) reverse Boudouard reactor integrated with green methanol synthesis; (c) biomass gasification process.

We investigate two scenarios (Figure 1) that combine different approaches for green methanol production. In order to ensure a fair comparison of the scenarios, we propose a system expansion approach. We evaluate the production of green methanol, H_2_ from biomass gasification (from which biochar is a byproduct) and the potential usage of biochar as a fuel for industrial high-temperature heat. The first scenario (Figure 1a), used as the base case for this study, is based on the optimized green methanol process investigated by González Garay et. al^7^ (Figure 2a), while the second scenario (Figure 1b) is built by integrating the base scenario with the RB reaction section (Figure 2b).

The base case corresponds to synthesis of green methanol by direct air captured (DAC) CO_2_ hydrogenation (Figure 2a). The CO_2_ feed (25 °C and 1 bar) is compressed to 50 bar in three compressors in series with intercooling to 40 °C. In parallel, H_2_ (25 °C and 30 bar) is also compressed to 50 bar and mixed with the CO_2_ stream with a molar H_2_/CO_2_ ratio close to three. The mixture is then heated up to the reaction temperature (228 °C) and fed to a plug flow reactor where the reactions take place adiabatically. The kinetic model is adapted from Busche and Froment.^27^ After the reaction section, the stream is cooled down to 45 °C before entering a flash separator where most of the unreacted syngas is recovered. The gas stream is recycled after a 0.1% purge, recompressed to compensate for the 5 bar pressure drop of the reactor and mixed with the feed H_2_/CO_2_ stream. The liquid stream exiting the flash separator is decompressed to 1.4 bar before entering a second phase separator where the remaining unreacted gases are removed, mixed with the purged fraction of the first separator and sent to a furnace where they are combusted with air. The liquid product is heated up to saturation temperature and sent to a distillation column. The bottom product of the column is considered wastewater, while the top products (liquid and vapor) are mixed and cooled down as the final methanol product with 99.8 wt %.

The second scenario (Figure 2b) studies the integrated RB reaction with green methanol synthesis. Here, CO_2_ is compressed to 55 bar and mixed with renewable solid carbon (i.e., biochar, a common byproduct of biomass gasification and pyrolysis that is often combusted) and heated up to the reaction temperature (2000 °C). In the RB reactor, solid carbon and CO_2_ react to form CO isothermally. The relationship between reaction temperature, conversion and reactor volume is modeled following a standard volume reaction model (VRM)^28^ (Supporting Information Section F). After this step, a pressure drop of 5 bar is assumed due to the potential removal of residual biochar dust from the mixture of CO and unreacted CO_2_ via a cyclone. Then, the stream is mixed with the fresh H_2_ and cooled down to the methanol reaction temperature. We note that CO_2_ used in this step is obtained from DAC, the same as the unintegrated green methanol process.

Biochar production is modeled in Apen Plus v12 based on the gasification of wood chips for H_2_ generation (Figure 2c).^29^ The biomass, with a low initial moisture content of 8.4 wt %, is directly gasified at 700 °C using a dry biomass-to-steam ratio of 0.5. A fraction of the biochar produced (c.a. 75%) is used as fuel in the gasifier, while the rest is obtained as a byproduct. The syngas stream, after the removal of sulfonated (H_2_S) components, enters a water gas shift (WGS) reactor to maximize H_2_ production. The H_2_ and CO_2_ mix is sent to a pressure swing adsorption (PSA) where 95% of the H_2_ at very high purity (99.999%) is obtained as the main product. The CO_2_ is then compressed to 110 bar and sent to storage. Although this biogenic CO_2_ could be a good candidate for methanol synthesis and/or the RB reaction, we note here that the biomass gasification process and the green methanol process are assumed to be delocalized, hence, making the integration unappealing. A more detailed description can be found in **Section A** of the Supporting Information.

### Environmental Assessment

We perform the LCA following the ISO14040/44 framework.^30^ The goal of the LCA is to compare two approaches to producing green methanol: the standard CO_2_ hydrogenation process and one integrating the former with the RB reaction. We define a functional unit (FU) of 1.00 kg of green methanol, 0.41 kg of biogenic H_2_ and 4.88 MJ of high-temperature industrial heating adopting a cradle-to-gate scope following a cutoff attributional approach. We compute the life cycle inventory (LCI) of all the inputs and outputs linked to green methanol synthesis and biomass gasification and industrial heating generation (i.e., feedstocks, emissions, and waste). The background system is modeled by combining the material and energy flows in the process simulations with literature information for electrolytic H_2_^31^ and DAC CO_2,_^32^ and data from the Ecoinvent v3.5^25^ database, which includes all materials, energy, waste and emissions associated with the production of raw materials, utilities and electricity. The life cycle impact assessment (LCIA) was computed using SimaPro v9.2.0.2 following the ReCiPe 2016 v1.13 method.^24^ We additionally performed a Monte Carlo analysis considering 2000 scenarios generated from Ecoinvent using the pedigree matrix to evaluate the probability of burden shifting in the carbon footprint and damage assessment categories.

### Economic Assessment

The total annualized cost (TAC) was estimated from the operating (OPEX) and capital investment (CAPEX) costs following standard methodologies.^26^ A more detailed description of the procedure and the economic parameters used in the calculations can be found in the Supporting Information (**Section C**). Additionally, we performed a sensitivity analysis of the green methanol synthesis cost varying the electrolytic H_2_ price, to identify the breakpoints where the base case and the integrated RB with green methanol become economically competitive with the fossil route.

## Results and Discussion

The simulation results are reported in the Supporting Information (**Section B**). Furthermore, additional environmental data (i.e., the individual contributions of the midpoints of the ReCiPe 2016 v1.13 methodology) can be found also in **Section B** of the Supporting Information. Here, we delve into the global warming and damage assessment impact results as well as the economic results.

### Environmental Results

**Figure 3:**
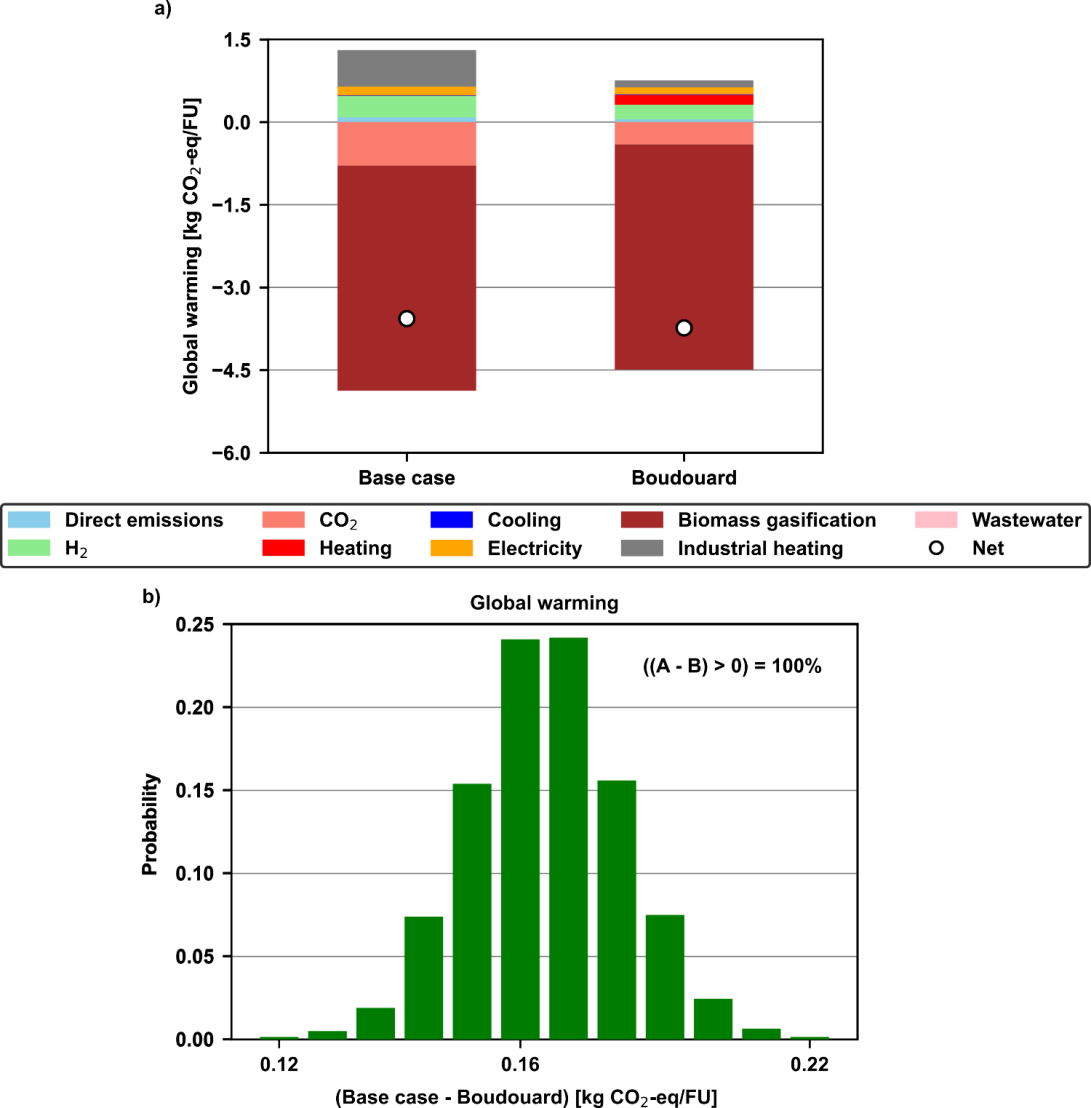
(a) Global warming impact per FU (1.00 kg of green methanol, 0.41 kg of biogenic H_2_ and 4.88 MJ of high-temperature industrial heating) of the assessed scenarios: base case standard green methanol synthesis via CO_2_ hydrogenation vs alternative green methanol synthesis using integrated biochar gasification with CO_2_ via the Boudouard reaction and green H_2_. The sum of the contributions of H_2_, CO_2_, heating, cooling, and electricity constitutes the green methanol synthesis process. The biomass gasification contribution includes the production of biogenic H_2_, biochar and captured CO_2_. The industrial heating contribution corresponds to the emissions associated with producing high-temperature heating for both scenarios. (b) Monte Carlo simulation results of the difference between the base case (A) minus the Boudouard scenario (B). A negative result implies burden shifting since the impact of A is lower than B.

As depicted in Figure 3a, the integration of green methanol with the RB reaction proves beneficial to the net carbon footprint of the expanded system with respect to the base scenario by 5%. This improvement is mainly attributed to the more efficient use of biochar as a carbon source for the RB reaction. Integrating this alternative raw material results in a lower electrolytic H_2_ and DAC CO_2_ demand in green methanol synthesis and, additionally, replacing biochar with wind electrolytic H_2_ in high-temperature heating applications further decreases CO_2_ emissions. More specifically, the biomass gasification negative contribution dominates the total impact of the expanded system, mainly due to the biogenic CO_2_ captured during the process of H_2_ production. In the base case, this contribution is followed by the use of biochar in industrial heating and finally, the net methanol synthesis, i.e., the DAC CO_2_ and electrolytic H_2_ raw materials, the electricity and utilities consumed in compression and the direct emissions of the process. In contrast, for the Boudouard scenario, the second most important contribution after biomass gasification is green methanol synthesis. This shift stems from the reduced DAC CO_2_ consumption required for methanol synthesis when using biochar as a carbon source (reaction 4), which increases the overall impact of the synthesis. Furthermore, the additional heating required for the endothermic and high-temperature RB reaction balances the reduced impact of consuming less H_2_ in the synthesis, yielding an overall more carbon-intensive green methanol synthesis than the base case scenario. However, it is worth noting that the biochar production contribution is included in the biomass gasification process, and with a different scope, that is, without a system expansion, the green methanol synthesis impact of the Boudouard scenario would drastically decrease.

Figure 3b shows the uncertainty analysis of these results as the impact of the base case scenario (A) minus the impact of the Boudouard scenario (B) for 2000 different backgrounds computed using the pedigree matrix of the Ecoinvent database. As depicted, 100% of the simulations yield positive differences, which means that the impact of the base case is always higher than the alternative scenario. Hence, we can affirm that, statistically, the Boudoaurd scenario outer performs the base case regarding the climate change impact.

**Figure 4:**
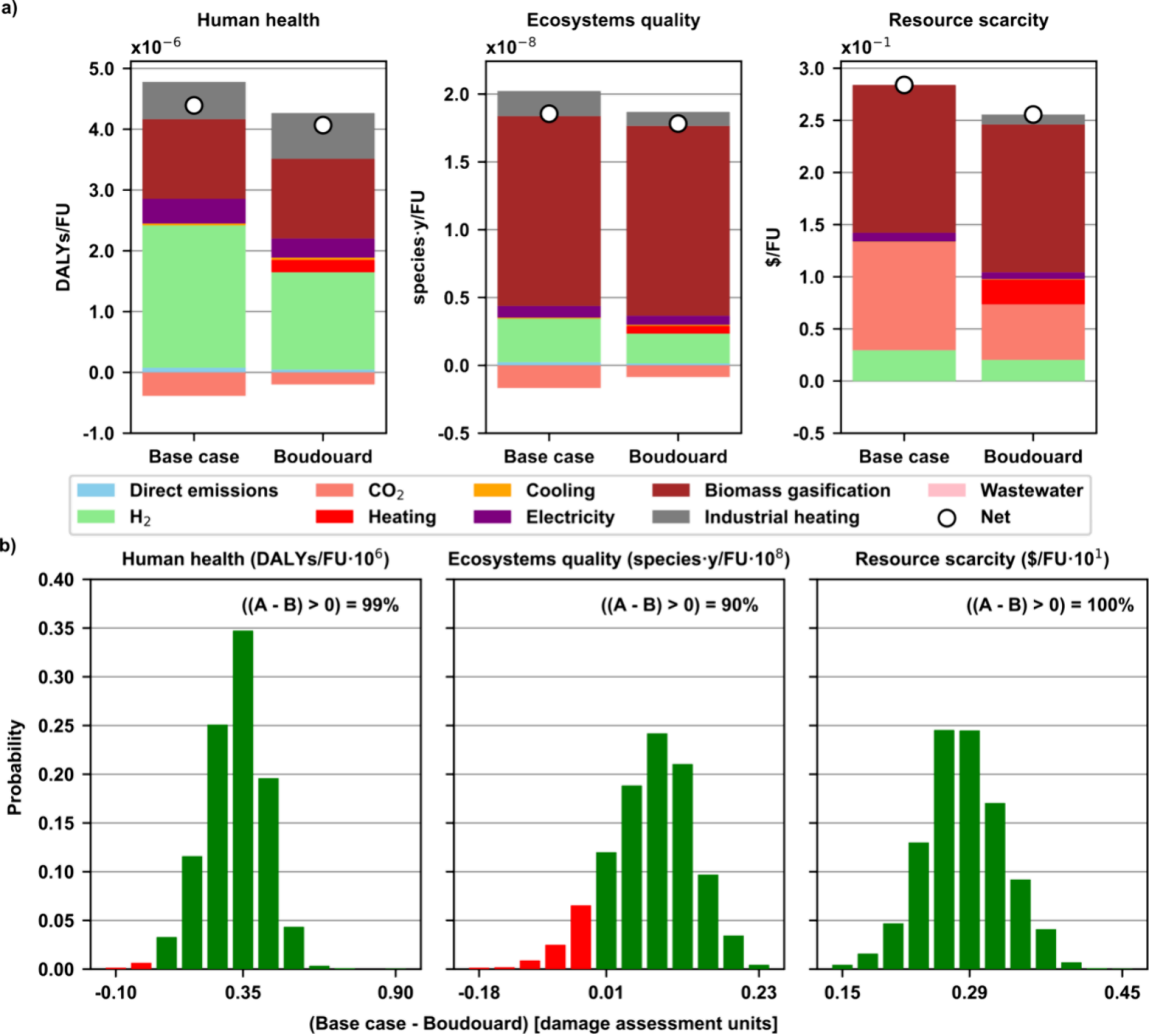
(a) System expansion end point impact results per FU (1.00 kg of green methanol, 0.41 kg of biogenic H_2_ and 4.88 MJ of high-temperature industrial heating): base case standard green methanol synthesis via CO_2_ hydrogenation (A) vs alternative green methanol synthesis using integrated biochar gasification with CO_2_ via the Boudouard reaction and green H_2_ (B). The sum of the contributions of H_2_, CO_2_, heating, cooling and electricity constitutes the green methanol synthesis process. The biomass gasification contribution includes the production of biogenic H_2_, biochar and captured CO_2_. The industrial heating contribution corresponds to the emissions associated with producing high-temperature heating for both scenarios. (b) Monte Carlo simulation results of the difference between the base case (A) minus the Boudouard scenario (B). A negative result implies burden shifting since the impact of A is lower than B.

Broadening the scope to other environmental metrics (Figure 4), we can see that for the end points (i.e., the aggregation of all 18 midpoint impacts of the ReCiPe 2016 into human health, ecosystems quality and resource scarcity, Figure 4a), the green methanol integrated with the RB expanded system performs better than the standard unintegrated base case system. That is, no burden shifting is detected in any of the evaluated impact metrics (Figure 4b). More specifically, the highest decrease is observed in resource scarcity (10%), followed by human health (7%), and finally, ecosystems quality (4%). This decrease is mainly attributed to the use of biochar as raw material in green methanol synthesis instead of fuel in high-temperature industrial heating (human health and ecosystems quality), and the DAC CO_2_ consumption reduction (resource scarcity). For the latter result, it is worth noting that capturing CO_2_ from the air reduces the global warming and the damage to human health and ecosystems, yet its heavy utility consumption (i.e., natural gas heating and grid electricity) negatively impacts resources.

Regarding the uncertainty analysis (Figure 4b), the results of the Monte Carlo simulation show that for human health and resource scarcity there is a 99 and 100% probability of the Boudouard scenario (B) outperforming the base case (A), or what is the same, the probability of occurrence of burden shifting is virtually zero. In contrast, ecosystems quality shows a 10% probability of burden shifting. All in all, due to the low occurrence of negative (A – B) results, we can conclude that in the overall system, there is a negligible probability of burden shifting.

### Economic Results

**Figure 5:**
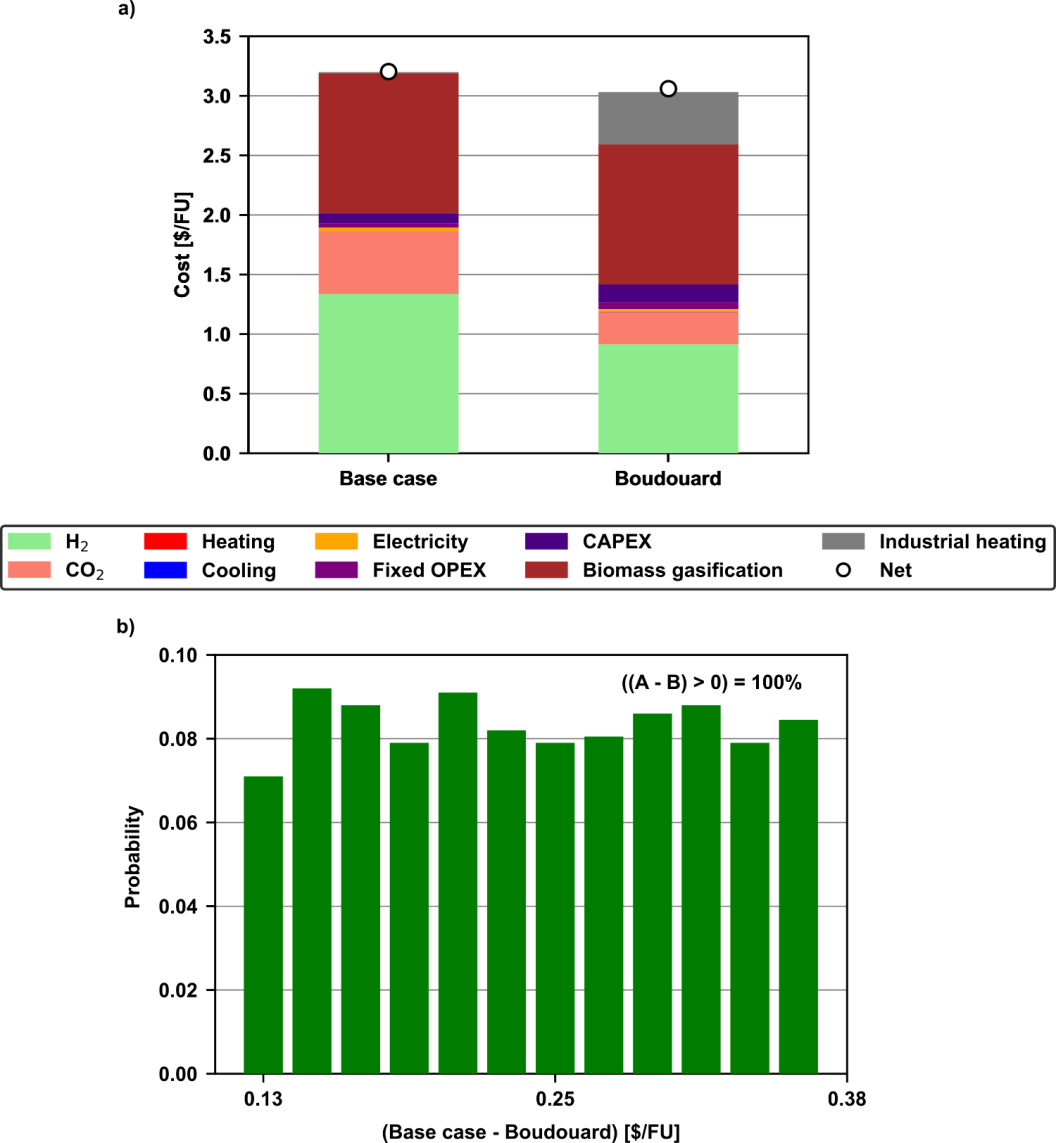
(a) Expanded system production cost comparison per FU (1.00 kg of green methanol, 0.41 kg of biogenic H_2_ and 4.88 MJ of high-temperature industrial heating) of the CO_2_ hydrogenation (base case) with the integrated green methanol with the RB reaction (Boudouard). The sum of the contributions of H_2_, CO_2_, heating, cooling, electricity, fixed OPEX, and CAPEX constitutes the green methanol synthesis process. The biomass gasification contribution includes the production of biogenic H_2_, biochar, and captured CO_2_. The industrial heating contribution corresponds to the emissions associated with producing high-temperature heating for both scenarios (base case scenario using the biochar from the gasification and Boudouard scenario using electrolytic H_2_). (b) Monte Carlo uncertainty analysis of the economic results varying H_2_ (5.23–8.48 $/kg) and CO_2_ (0.269–0.623 $/kg) prices considering a uniform distribution for each element in the range.^33^ A corresponds to the base case scenario and B to the Boudouard scenario. A positive result from A – B represents an economic advantage.

As shown in Figure 5a, coupling an RB reactor with green methanol synthesis results in a 5% overall cost decrease in the expanded system compared with the base case direct CO_2_ hydrogenation. The main contributor to this reduction is the lower demand for DAC CO_2_ in the synthesis.

In general, green methanol synthesis (that is, the contributions of H_2_, CO_2_, utilities, fixed OPEX and CAPEX) dominates the cost of the base case expanded system, with 63% of the total contribution. This is followed by biomass gasification with 37% and industrial heating, with a negligible contribution, which is exclusively attributed to the furnace CAPEX since the fuel, i.e., biochar, is included in the biomass gasification process contribution. In contrast, the Boudouard scenario expanded system shows a green methanol total contribution of 46%, with biomass gasification and industrial heating at 39 and 15%, respectively. This appreciable change in the shares originates from the resource relocation of H_2_ and biochar. Using the RB reaction for green methanol synthesis reduces H_2_ demand by 32% (from 0.19 to 0.13 kg/kg of methanol) which drastically affects the production cost of green methanol. However, the H_2_ saved in this process is used in industrial heating instead of the biochar, which in the end balances the total cost associated with H_2_ usage. Furthermore, DAC CO_2_ consumption is reduced by 49%. These results suggest that selecting a different fuel rather than H_2_ for industrial heating, such as coal or natural gas, would drastically decrease the cost of the Boudouard expanded system, potentially increasing the economic gap between the two systems by up to 20%, albeit likely compromising the environmental.

The uncertainty analysis (Figure 5b) proves, once again, that the base case is outperformed by the Boudouard scenario. Here, the price of H_2_ (5.23 – 8.48 $/kg) and CO_2_ (0.269 – 0.623 $/kg), the most important contributors to green methanol synthesis cost, were varied assuming current trends.^33^ In essence, this analysis demonstrates that the costs of both systems are correlated, that is, H_2_ and CO_2_ are consumed at approximately the same rates. Hence, an increase or decrease in raw material price affects both scenarios proportionally, and the economic advantage of the Boudouard scenario is always predominant.

**Figure 6:**
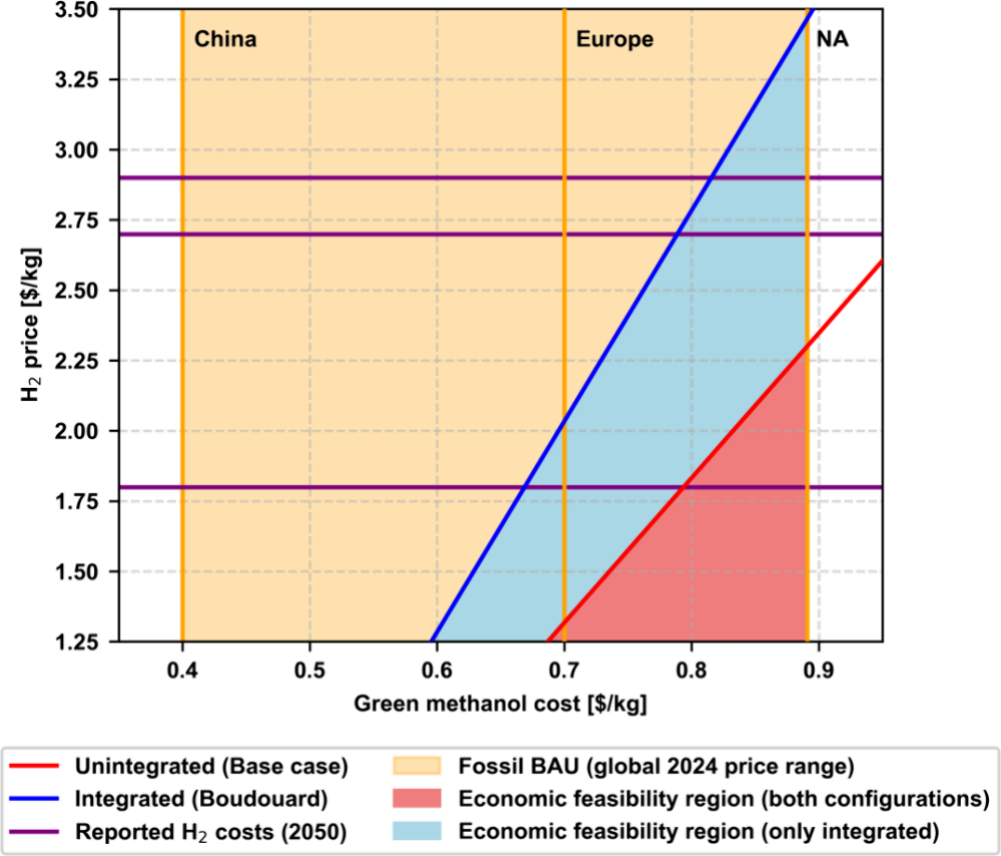
Standard CO_2_ hydrogenation (unintegrated (base case)) and green methanol integrated with RB (integrated (Boudouard)) production cost with variable H_2_ prices. Comparison with the fossil business-as-usual (fossil BAU) methanol first quarter of 2025 contract price range in China, Europe and North America (NA).^35^ The colored zones indicate the range of global fossil methanol prizes (all), the RB integrated green methanol economic feasibility region (blue and red), and the standard green methanol economic feasibility region (red). Recently published projections of future H_2_ minimum costs in 2050 are shown as horizontal lines: 2.9,^37^ 2.7,^38^ and 1.8 $/kg.^39^

As discussed based on the results in Figure 5 and supported by many other works,^7−9^ the production cost of green methanol is heavily dependent on the electrolytic H_2_ price. For this reason, we perform a sensitivity analysis to assess the impact of this parameter on the economic competitiveness of green methanol. For this evaluation, we decouple green methanol production from the expanded system assuming a cost of biochar of 0.16 $/kg.^34^ In Figure 6, we show how the green methanol cost varies for the base case scenario (standard green methanol from CO_2_ hydrogenation) and the Boudouard scenario (green methanol integrated with RB) with the H_2_ price, and also provide the current range (0.40 to 0.89 $/kg in the first quarter of 2025)^35^ of fossil methanol prices. As depicted in the figure, the integrated green methanol with RB would require a minimum price of 3.5 $/kg of electrolytic H_2_ to become competitive relative to the highest reported price of fossil methanol (0.89 $/kg), considering a projected cost of CO_2_ of 0.20 $/kg.^32^ Meanwhile, the breakeven H_2_ price for the unintegrated configuration is 2.3 $/kg, i.e., due to the lower H_2_ requirements, the integrated RB configuration reaches economic competitiveness with H_2_ priced ∼ 50% higher than the standard green methanol. In contrast, looking at the European methanol market, the RB configuration would become cost competitive with H_2_ priced at 2.0 $/kg, while the traditional green methanol unintegrated process would require a price of 1.3 $/kg of H_2_. This decrease seems still far from current green H_2_ prices, however, it is worth noting that despite the conservative value of 6.23 $/kg^33^ of wind-powered electrolytic H_2_ we use in this study, costs as low as 3.45 $/kg have already been reported,^36^ with prospects to reduce the cost even more by 2050.^37−39^ Furthermore, subsidies for green H_2_ of up to 3 $/kg were recently approved in the US Credit for production of clean hydrogen plan,^40^ which implies that under specific conditions our alternative scenario would already become competitive even considering the lower end of fossil methanol current prices.

## Conclusions

In this work, we studied the performance of the green methanol production process integrated with an RB reactor and compared it to the classic CO_2_ hydrogenation synthesis using process simulation and LCA. This synergistic combination results in a better economic and environmental performance than the standard green methanol evaluated under an expanded system that assumes alternative uses of the same resources. More specifically, integrating the RB reaction allows producing CO from CO_2_ and biochar, reducing the use of expensive H_2_. Moreover, biochar use as a chemical feedstock instead of fuel leads to decreased overall costs without compromising total environmental impacts on human health, ecosystems and resources. This integration leads to a 5% overall decrease in production cost and the use of byproduct biochar from woody biomass gasification as the solid carbon source results in no significant probability of burden shifting, with reductions of 4 – 10% in all damage assessment metrics. As methanol is a bulk chemical with the potential to become a fuel in the future, these improvements could become particularly relevant for mitigating the economic burden of the sustainable transition.

Despite this win-win scenario when compared with the standard green methanol synthesis, the RB-integrated green methanol would still require an H_2_ price of 3.5 – 2.0 $/kg to currently become competitive with its fossil counterpart in North America and Europe. However, unintegrated CO_2_ hydrogenation green methanol needs an even more restrictive 2.3 – 1.3 $/kg to attain competitiveness. Hence, these results greatly close the gap toward a cost-competitive green methanol synthesis, and more so if we consider future improvements in the technology, such as more efficient electrolyzers or wind generators that can rapidly cut H_2_ prices in the following years. Moreover, regulations to subsidize H_2_ costs could already make this new green methanol configuration economically competitive.

Going beyond green methanol, we stress the importance of evaluating integrated configurations for chemical synthesis to potentially enhance the economic and environmental competitiveness of green routes. More specifically, our results suggest that in an era where electrolytic H_2_ is a priced and limited resource, there are novel integrated routes to direct CO_2_ hydrogenation that can make a more efficient use of it. Moreover, with this work, we aim to encourage the experimental development of alternative reaction pathways, such as the reverse Boudoaurd reaction, as they have the potential to help make the chemical industry more sustainable.

## Supporting Information Available

Design and results of the simulations (Section A), additional results (Section B), heat integration (Section C), economic assessment (Section D) and results (Section E), reactor volume estimation (Section F), and temperature effect on the sustainability results (Section G) (PDF)

## Abbreviations

BAU: business-as-usual
CAPEX: capital investment cost
CO_2_-eq: CO_2_ equivalent
DAC: direct air capture
FU: functional unit
LCA: life cycle assessment
LCI: life cycle inventory
LCIA: life cycle impact assessment
OPEX: operating cost
RB: reverse Boudouard
RWGS: reverse water gas shift
SMR: steam methane reforming
TAC: total annualized cost
WGS: water gas shift
SHC: specific H_2_ consumption
