# Supplementary material for: Integrating the Reverse Boudouard Reaction for a More Efficient Green Methanol Synthesis from CO2 and Renewable Energy
Source: ACS Sustain Chem Eng. 2025 May 8;13(19):7088–97. doi: 10.1021/acssuschemeng.5c01021 (PMC12093377; doi:10.1021/acssuschemeng.5c01021)
Supplement: Supplementary file 1 — sc5c01021_si_001.pdf [file sc5c01021_si_001.pdf]

Integrating the Reverse Boudouard Reaction for a More Efficient Green Methanol Synthesis from CO_2 and Renewable Energy

Juan D. Medrano-García[*,†,††], *Marina T. Chagas*[†] *and Gonzalo-Guillén-Gosálbez*[*,†,††]

[†] Institute for Chemical and Bioengineering, Department of Chemistry and Applied Biosciences, ETH Zurich, Vladimir Prelog Weg 1, 8093 Zurich, Switzerland.

[††] NCCR Catalysis, Switzerland

[*] Corresponding author.

E-mail address: juan.diego.medrano@chem.ethz.ch.

E-mail address: gonzalo.guillen.gosalbez@chem.ethz.ch.

This document includes additional material to the content presented in the main article. Here we report the methodology and design of the simulations, the life cycle inventories, further environmental results and the economic analysis description and results.

Number of pages: 44

Number of figures: 6

Number of tables: 30

A. Process simulations

We developed three process simulations to evaluate green methanol synthesis under different scenarios. These scenarios consist of the standard CO_2 hydrogenation (base case) and the integration of the base case with a reverse Boudouard (RB) reaction operating at 2000 ºC. The simulated plant annual capacities were 485 and 470 kt/y, respectively, for an 8000 hours operation per year. We use Aspen HYSYS v11 with the Peng-Robinson thermodynamic model for the compression and reaction sections of the simulations, and the NRTL model for the separation. We consider all heating coming from natural gas combustion at 90% efficiency.

The third simulation consists of a biomass gasification process to produce H_2 and biochar as a by-product. This simulation was developed in Aspen Plus v11 using the Peng-Robinson thermodynamic model.

We compute the minimum utility consumption of all simulations with Aspen Energy Analyzer. The inventories resulting from the material and energy flows of the simulations are presented in **Section B**. Here we explain the process simulations in detail.

A1. Green methanol (base case scenario)

The standard CO_2 hydrogenation process for green methanol production (**Figure S1**) is based on the results of González-Garay et al.[1]

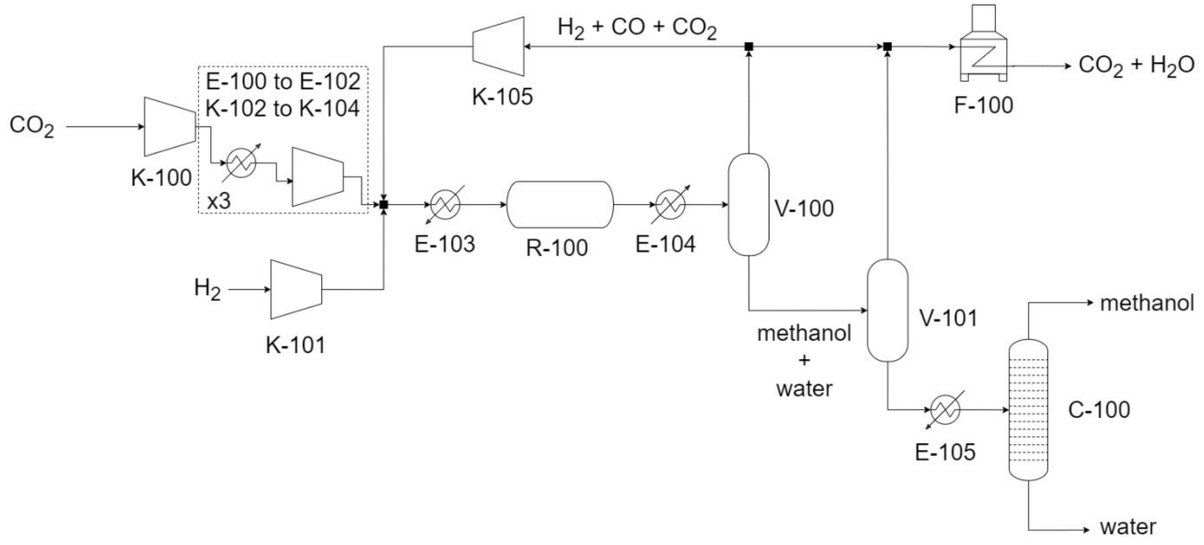

Figure S1. Green methanol process flowsheet scheme.

Here, CO_2 (25 ºC, 1 bar) and H_2 (25 ºC, 30 ºbar) are compressed to 50 bar and mixed before entering the adiabatic reactor (51 m^3, 5 bar pressure drop, 15% CO_2 conversion) at 228 ºC (R-100). The kinetic model used for the reaction system was adapted from Busche and Froment.[2] The outlet of the reactor is cooled down to 40 ºC and sent to a first flash separator (V-100), where most of the unreacted CO_2, H_2 and byproduct CO are recompressed to 50 bar and mixed with the fresh CO_2 and H_2 feed after a small purge (0.23%). The liquid product of the flash is sent to a second flash unit (V-101) after depressurizing to 1.8 bar. The vapor is mixed with the purged and combusted with air, while the liquid is heated up to 80 ºC and sent to the distillation column (C-100). Here, water is obtained in the bottoms while the methanol product (99.9 mol.%) is recovered as the distillate. The column is designed with 52 ideal stages (80 ºC feed at the 28th stage from the top), a reflux ratio of 1.25. The reboiler operates at 1.4 bar and 109 ºC and consumes 2.79 MJ/kg of methanol. The condenser operates at 1 bar and 64 ºC and requires 2.68 MJ/kg. The methanol molar recovery is 99% with a 99.9 wt.% purity. The overall CO_2 to methanol conversion was 95%.

A2. Green methanol with integrated reverse Boudouard reaction (Boudouard scenario)

The RB reactor was integrated with the green methanol process operating at 2000 ºC and 55 bar. This temperature was chosen after evaluating the energy and raw material consumption trade-offs derived from higher CO_2 conversions in the RB reactor (Section E). The design of this section is shown in **Figure S2**. First, CO_2 (25 ºC, 1 bar) is compressed to 55 bar with intercooling to 40 ºC in three stages. The stream is mixed with solid carbon (biochar at 25 ºC and 1 bar) heated up to the reaction temperature (2000 ºC) and sent to an isothermal conversion reactor (R-200), modeled the results of a kinetic model (89% conversion and 100 m^3)[3] (more details in Section E). Then, unreacted biochar is separated from the gas phase and recycled back to the inlet of the reactor after a 5 bar pressure drop, while the unreacted CO_2 and the product CO are sent to the green methanol synthesis loop (**Figure S1**). We assume that all biochar is separated and that the cost of doing so is negligible. The overall and per pass conversions in the methanol synthesis reactor for this configuration are 97 and 8%, respectively.

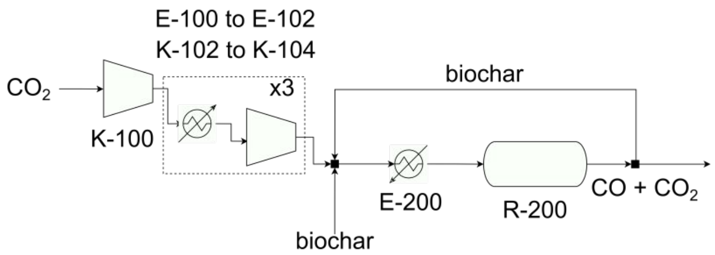

Figure S2. Reverse Boudouard section scheme.

A3. Biomass gasification for hydrogen production with carbon capture

The biomass gasification process was simulated in Aspen Plus v12 based on literature. (**Figure S3**).[4] The model uses the Peng-Robinson equation of state with Boston-Matias modifications to estimate the thermodynamic properties.[5] The biomass, assumed to be birch bark, is specified as a non-conventional component and the HCOALGEN and DCOALIGT property models are used to estimate the biomass enthalpy of formation, heat capacity and density. The proximate, ultimate and sulfur analyses (**Table S1**) were adapted from the work of Vassilev et al.[6]

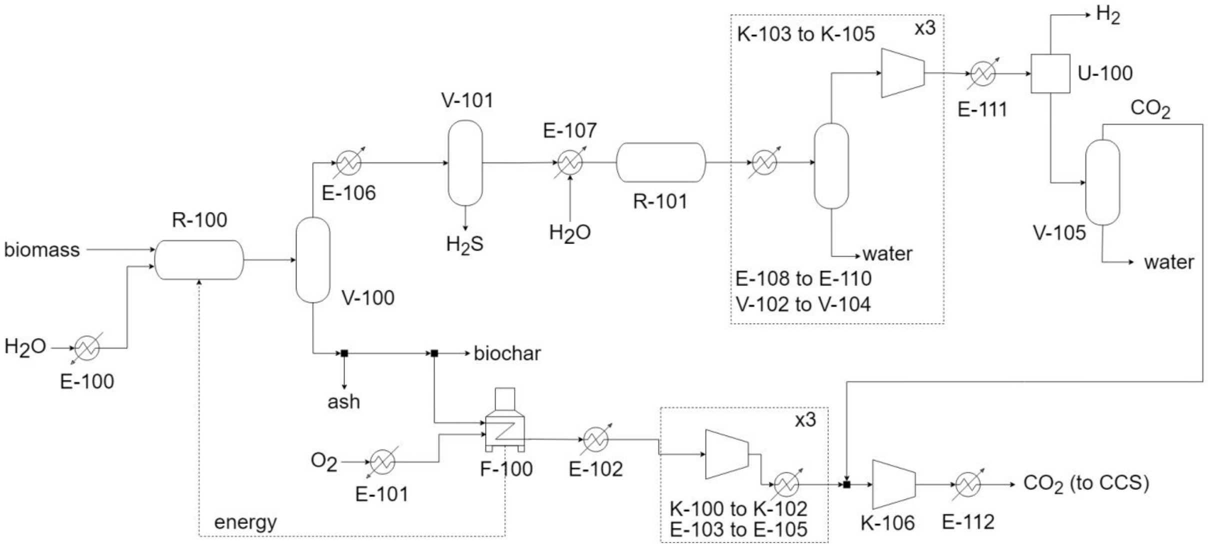

Figure S3. Biomass gasification process flowsheet scheme.

Due to the low moisture content of the feedstock, no drying was assumed to be necessary. The biomass is decomposed into its conventional components (C, O_2, H_2, N_2, S, H_2O, char, and ash) in a yield reactor operating at 700 °C and 1 bar. This is done by specifying the yield distribution taking into consideration the ultimate analysis of biomass and its moisture content.[7] In our model, tar formation is not considered.[4] Char is assumed to be pure carbon, and the amount produced in the gasifier is equal to the fixed carbon in the biomass.

Table S1. Birch bark proximate, ultimate and sulfur analyses.

	Proximate analysis	Ultimate analysis	Sulfur analysis
**Component**	**Value**	**Value**	**Value**
Moisture	8.40	-	-
Fixed carbon (FC)	19.43	-	-
Volatile matter (VM)	78.49	-	-
Ash	2.07	2.07	-
Carbon	-	55.82	-
Hydrogen	-	6.56	-
Nitrogen	-	0.49	-
Chlorine	-	-	-
Sulfur	-	0.10	-
Oxygen	-	34.96	-
Pyritic	-	-	-
Sulfate	-	-	0.10
Organic	-	-	-

The stream containing the conventional components then enters the gasifier (R-100), modeled as an isothermal Gibbs reactor operating at 700 °C and 1 bar. Steam, the selected gasifying agent, is preheated to 450 °C before entering the gasifier. The amount of steam introduced in the gasifier was determined so that a steam-to-biomass ratio of two was obtained.[8]

After the gasifier, char and ash are separated from the syngas in a separation unit (V-100). It is assumed that they can be completely separated one from the other, resulting in two pure solid streams. A fraction of the char is converted into pure carbon in a stoichiometric reactor and combusted to provide heat for the gasifier, whereas the remaining char is cooled down to 40 °C and obtained as a by-product. The fraction of char to be combusted (75.3 wt.%) was set so that the gasification took place isothermally. The furnace (F-100) is modeled as a Gibbs reactor operating at 750 °C, and stoichiometric oxygen is preheated to 450 °C before entering the reactor. The flue gas from the char combustion, containing almost 100% pure CO_2, is cooled down to 40 °C and then compressed to 30 bar in a series of three compression stages with intercooling to 40 °C.

As the formation of inorganic contaminants also occurs in the gasifier, syngas cleaning is carried out to remove hydrogen sulfide (H_2S). The syngas is cooled down to 450 °C before entering a separation unit (V-101), in which 99% of the contaminant is assumed to be removed[9].

The syngas stream leaving the cleaning section is cooled down to 250 °C and fed along with additional steam, preheated to 250 °C, to a water gas shift (WGS) reactor (R-101), modeled as a Gibbs reactor, in order to maximize the amount of hydrogen in the syngas. The outlet stream is cooled down to 40 °C and sent to a flash separator to remove most of the water (V-102). The vapor stream is compressed to 30 bar in a series of three compressors with intercooling to 40 °C and water removal.

The hydrogen rich syngas stream is then fed to a pressure swing adsorption (PSA) unit (U-100), modeled as a component separator with a defined split ratio for hydrogen[10]. The unit is assumed to operate at 40 °C and 30 bar. A recovery of 95% and purity of 99.99% is assumed for hydrogen[11]. The off-gas stream, consisting of 99.13 wt.% CO_2, is mixed to the compressed CO_2 stream from char combustion and the resulting stream is further compressed to 110 bar for CO_2 storage.

B. Life cycle assessment (LCA)

In this section, we present the main results of the simulations as the net material and energy streams per functional unit, including the standard CO_2 hydrogenation process and the reverse Boudouard (RB) integrated configuration. Then, we show the ReCiPe 2016 v1.13 midpoints, including the sensitivity analysis for the global warming impact, and endpoints[12] results for all the studied scenarios.

B.1. Life cycle inventories (LCIs)

In this section, we present the main results of the simulations (net material and energy streams per functional unit of product) in the form of LCIs (Tables S2 – S4). Furthermore, Tables S5 – S6 represent the expanded systems inventories for the evaluated scenarios. The Cu-ZnO-Al_2O_3 catalyst was not considered in the environmental calculations, as its contribution is usually negligible and hence it is often omitted in LCA studies.[1]

Table S2. CO_2 hydrogenation to green methanol LCI.

Functional unit: 1 kg of green methanol		
**Input**	**Amount**	**Units**
Hydrogen (from wind electrolysis)[13]	$1.94 \cdot 10^{-1}$	kg
Carbon dioxide (from direct air capture)[14]	1.45	kg
Air (natural resource)	$4.28 \cdot 10^{-1}$	kg
Heating (from natural gas)	0.00	MJ
Cooling (water 20 to 25 ºC)[15]	6.20	MJ
Electricity (high voltage)	$2.99 \cdot 10^{-1}$	kWh
**Output**		
Green methanol	1.00	kg
Carbon dioxide (emission to air)	$8.30 \cdot 10^{-2}$	kg
Carbon monoxide (emission to air)	$3.65 \cdot 10^{-9}$	kg
Hydrogen (emission to air)	$1.92 \cdot 10^{-10}$	kg
Nitrogen (emission to air)	$3.28 \cdot 10^{-1}$	kg
Oxygen (emission to air)	$6.32 \cdot 10^{-2}$	kg
Water (emission to air)	$3.58 \cdot 10^{-2}$	kg
Methanol (emission to water)	$9.91 \cdot 10^{-4}$	kg
Water (emission to water)	$5.73 \cdot 10^{-4}$	m^3

Table S3. Reverse Boudouard integrated with green methanol LCI.

Functional unit: 1 kg of green methanol		
**Input**	**Amount**	**Units**
Hydrogen (from wind electrolysis)[13]	$1.33 \cdot 10^{-1}$	kg
Carbon dioxide (from direct air capture)[14]	$7.46 \cdot 10^{-1}$	kg
Biochar from biomass gasification (**Table S4**)	$1.81 \cdot 10^{-1}$	kg
Air (natural resource)	$5.87 \cdot 10^{-1}$	kg
Heating (from natural gas)	4.65	MJ
Cooling (water 20 to 25 ºC)[15]	40.1	MJ
Electricity (high voltage)	$2.32 \cdot 10^{-1}$	kWh
**Output**		
Green methanol	1.00	kg
Carbon dioxide (emission to air)	$4.94 \cdot 10^{-2}$	kg
Nitrogen (emission to air)	$4.50 \cdot 10^{-1}$	kg
Oxygen (emission to air)	$9.13 \cdot 10^{-2}$	kg
Water (emission to air)	$3.56 \cdot 10^{-2}$	kg
Water (emission to water)	$2.93 \cdot 10^{-5}$	m^3

Table S4. Biomass gasification of birch bark with carbon capture LCI.

**Functional unit: 1 kg of biochar***		
**Input**	**Amount**	**Units**
Wood chips, wet, dry mass, birch	20.9	kg
Water (tap)	7.81	kg
Oxygen	8.14	kg
Cooling (water 20 to 25 ºC)[15]	67.7	MJ
Electricity (high voltage)	9.29	kWh
**Output**		
Biogenic hydrogen	2.29	kg
Biochar	1	kg
Carbon dioxide (captured at 110 bar)	34.8	kg
Carbon dioxide (emission to water)	$3.90 \cdot 10^{-5}$	kg
Carbon monoxide (emission to water)	$3.88 \cdot 10^{-8}$	kg
Hydrogen sulfide (emission to water)	$7.10 \cdot 10^{-10}$	kg
Ammonia (emission to water)	$3.93 \cdot 10^{-33}$	kg
Nitrogen (emission to water)	$1.33 \cdot 10^{-5}$	kg
Methane (emission to water)	$5.56 \cdot 10^{-7}$	kg
Sulfur (emission to water)	$6.55 \cdot 10^{-13}$	kg
Water (emission to water)	5.81	kg

*not allocated

Table S5. High temperature heating fueled with biochar LCI.

Functional unit: 1 MJ of heating at 900 ºC		
**Input**	**Amount**	**Units**
Biochar from biomass gasification (**Table S3**)	$3.71 \cdot 10^{-2}$	kg
**Output**		
Heating	1	MJ
Carbon dioxide (emission to air)	$1.36 \cdot 10^{-1}$	kg

Table S6. High temperature heating fueled with hydrogen LCI.

Functional unit: 1 MJ of heating at 900 ºC		
**Input**	**Amount**	**Units**
Hydrogen (from wind electrolysis)[13]	$1.28 \cdot 10^{-2}$	kg
**Output**		
Heating	1	MJ
Water (emission to air)	$1.49 \cdot 10^{-3}$	kg
Water (emission to water)	$9.96 \cdot 10^{-2}$	kg

Table S7. Base case scenario expanded system LCI.

Functional unit: 1 kg of green methanol, 0.41 kg biogenic H_2 and 4.88 MJ of high temperature heat		
**Input**	**Amount**	**Units**
Green methanol synthesis (**Table S2**)	1	kg
Industrial heat from biochar (**Table S5**)*	4.88	MJ

*includes the biomass gasification process and thus the production of 0.41 kg of biogenic H_2

Table S8. Reverse Boudouard (RB) scenario expanded system LCI.

Functional unit: 1 kg of green methanol, 0.41 kg biogenic H_2 and 4.88 MJ of high temperature heat		
**Input**	**Amount**	**Units**
Green methanol synthesis using RB (**Table S3**)*	1	kg
Industrial heat from hydrogen (**Table S6**)	4.88	MJ

*includes the biomass gasification process and thus the production of 0.41 kg of biogenic H_2

B.2. Midpoint results

In this section, we present the midpoint results of all the studied scenarios.

Table S9. ReCiPe 2016 v1.13 midpoint results for the base case scenario [impact/functional unit].

Impact category	Unit	Total	Green methanol	Biomass gasification	High temperature heating
Global warming	kg CO_2-eq	$-3.57\cdot10^{0}$	$-1.47\cdot10^{-1}$	$-4.08\cdot10^{0}$	$1.25\cdot10^{-1}$
Stratospheric ozone depletion	kg CFC11-eq	$1.42\cdot10^{-6}$	$4.27\cdot10^{-7}$	$9.97\cdot10^{-7}$	$7.56\cdot10^{-8}$
Ionizing radiation	kBq Co-60-eq	$3.36\cdot10^{-1}$	$6.93\cdot10^{-2}$	$2.67\cdot10^{-1}$	$8.63\cdot10^{-3}$
Ozone formation, Human health	kg NOx-eq	$9.30\cdot10^{-3}$	$2.31\cdot10^{-3}$	$6.99\cdot10^{-3}$	$3.83\cdot10^{-4}$
Fine particulate matter formation	kg PM2.5-eq	$8.03\cdot10^{-3}$	$2.39\cdot10^{-3}$	$6.02\cdot10^{-3}$	$3.94\cdot10^{-4}$
Ozone formation, Terrestrial ecosystems	kg NOx-eq	$9.46\cdot10^{-3}$	$1.73\cdot10^{-3}$	$7.07\cdot10^{-3}$	$3.97\cdot10^{-4}$
Terrestrial acidification	kg SO_2-eq	$1.65\cdot10^{-2}$	$5.02\cdot10^{-3}$	$1.15\cdot10^{-2}$	$9.09\cdot10^{-4}$
Freshwater eutrophication	kg P-eq	$2.20\cdot10^{-3}$	$8.19\cdot10^{-4}$	$1.38\cdot10^{-3}$	$2.06\cdot10^{-4}$
Marine eutrophication	kg N-eq	$1.56\cdot10^{-4}$	$5.99\cdot10^{-5}$	$9.62\cdot10^{-5}$	$1.40\cdot10^{-5}$
Terrestrial ecotoxicity	kg 1,4-DCB	$1.55\cdot10^{1}$	$1.31\cdot10^{0}$	$2.40\cdot10^{0}$	$4.07\cdot10^{0}$
Freshwater ecotoxicity	kg 1,4-DCB	$7.66\cdot10^{-1}$	$6.88\cdot10^{-1}$	$7.77\cdot10^{-2}$	$2.18\cdot10^{-1}$
Marine ecotoxicity	kg 1,4-DCB	$9.54\cdot10^{-1}$	$8.48\cdot10^{-1}$	$1.06\cdot10^{-1}$	$2.68\cdot10^{-1}$
Human carcinogenic toxicity	kg 1,4-DCB	$2.15\cdot10^{-1}$	$1.17\cdot10^{-1}$	$9.80\cdot10^{-2}$	$3.30\cdot10^{-2}$
Human non-carcinogenic toxicity	kg 1,4-DCB	$7.88\cdot10^{0}$	$4.04\cdot10^{0}$	$3.84\cdot10^{0}$	$1.21\cdot10^{0}$
Land use	m^2a crop-eq	$2.34\cdot10^{0}$	$2.90\cdot10^{-2}$	$2.31\cdot10^{0}$	$5.50\cdot10^{-3}$
Mineral resource scarcity	kg Cu-eq	$1.59\cdot10^{-2}$	$1.43\cdot10^{-2}$	$1.61\cdot10^{-3}$	$4.46\cdot10^{-3}$
Fossil resource scarcity	kg oil-eq	$1.16\cdot10^{0}$	$4.46\cdot10^{-1}$	$7.15\cdot10^{-1}$	$3.00\cdot10^{-2}$
Water consumption	m^3	$6.70\cdot10^{-2}$	$1.65\cdot10^{-2}$	$5.13\cdot10^{-2}$	$1.61\cdot10^{-3}$

Table S10. ReCiPe 2016 v1.13 midpoint results for the Boudouard scenario [impact/functional unit].

Impact category	Unit	Total	Green methanol	Biomass gasification	High temperature heating
Global warming	kg CO_2-eq	$-3.74 \cdot 10^{0}$	$2.23 \cdot 10^{-1}$	$-4.08 \cdot 10^{0}$	$6.63 \cdot 10^{-1}$
Stratospheric ozone depletion	kg CFC11-eq	$1.41 \cdot 10^{-6}$	$3.40 \cdot 10^{-7}$	$9.97 \cdot 10^{-7}$	0.00
Ionizing radiation	kBq Co-60-eq	$3.23 \cdot 10^{-1}$	$4.71 \cdot 10^{-2}$	$2.67 \cdot 10^{-1}$	0.00
Ozone formation, Human health	kg NOx-eq	$9.07 \cdot 10^{-3}$	$1.70 \cdot 10^{-3}$	$6.99 \cdot 10^{-3}$	0.00
Fine particulate matter formation	kg PM2.5-eq	$7.81 \cdot 10^{-3}$	$1.40 \cdot 10^{-3}$	$6.02 \cdot 10^{-3}$	0.00
Ozone formation, Terrestrial ecosystems	kg NOx-eq	$9.22 \cdot 10^{-3}$	$1.75 \cdot 10^{-3}$	$7.07 \cdot 10^{-3}$	0.00
Terrestrial acidification	kg SO_2-eq	$1.59 \cdot 10^{-2}$	$3.50 \cdot 10^{-3}$	$1.15 \cdot 10^{-2}$	0.00
Freshwater eutrophication	kg P-eq	$2.15 \cdot 10^{-3}$	$5.60 \cdot 10^{-4}$	$1.38 \cdot 10^{-3}$	0.00
Marine eutrophication	kg N-eq	$1.49 \cdot 10^{-4}$	$3.87 \cdot 10^{-5}$	$9.62 \cdot 10^{-5}$	0.00
Terrestrial ecotoxicity	kg 1,4-DCB	$1.54 \cdot 10^{1}$	$8.98 \cdot 10^{0}$	$2.40 \cdot 10^{0}$	0.00
Freshwater ecotoxicity	kg 1,4-DCB	$7.67 \cdot 10^{-1}$	$4.71 \cdot 10^{-1}$	$7.77 \cdot 10^{-2}$	0.00
Marine ecotoxicity	kg 1,4-DCB	$9.55 \cdot 10^{-1}$	$5.80 \cdot 10^{-1}$	$1.06 \cdot 10^{-1}$	0.00
Human carcinogenic toxicity	kg 1,4-DCB	$2.11 \cdot 10^{-1}$	$8.01 \cdot 10^{-2}$	$9.80 \cdot 10^{-2}$	0.00
Human non-carcinogenic toxicity	kg 1,4-DCB	$7.81 \cdot 10^{0}$	$2.76 \cdot 10^{0}$	$3.84 \cdot 10^{0}$	0.00
Land use	m^2a crop-eq	$2.34 \cdot 10^{0}$	$1.97 \cdot 10^{-2}$	$2.31 \cdot 10^{0}$	0.00
Mineral resource scarcity	kg Cu-eq	$1.59 \cdot 10^{-2}$	$9.82 \cdot 10^{-3}$	$1.61 \cdot 10^{-3}$	0.00
Fossil resource scarcity	kg oil-eq	$1.07 \cdot 10^{0}$	$3.25 \cdot 10^{-1}$	$7.15 \cdot 10^{-1}$	0.00
Water consumption	m^3	$6.50 \cdot 10^{-2}$	$1.21 \cdot 10^{-2}$	$5.13 \cdot 10^{-2}$	$-8.02 \cdot 10^{-4}$

B.3. Endpoint results

In this section, we present the endpoint results of the studied scenarios.

Table S11. ReCiPe 2016 v1.13 endpoint results for the base case scenario [impact/functional unit].

**Damage category**	**Unit**	**Total**	**Green methanol**	**Biomass gasification**	**High temperature heating**
Human Health	DALY	$4.39 \cdot 10^{-6}$	$2.47 \cdot 10^{-6}$	$1.31 \cdot 10^{-6}$	$6.13 \cdot 10^{-7}$
Ecosystems	species·yr	$1.86 \cdot 10^{-8}$	$2.70 \cdot 10^{-9}$	$1.40 \cdot 10^{-8}$	$1.85 \cdot 10^{-9}$
Resources	$	$2.84 \cdot 10^{-1}$	$1.42 \cdot 10^{-1}$	$1.42 \cdot 10^{-2}$	0

Table S.12 ReCiPe 2016 v1.13 endpoint results for the Boudouard scenario [impact/functional unit].

**Damage category**	**Unit**	**Total**	**Green methanol**	**Biomass gasification**	**High temperature heating**
Human Health	DALY	$4.07 \cdot 10^{-6}$	$2.01 \cdot 10^{-6}$	$1.31 \cdot 10^{-6}$	$7.52 \cdot 10^{-7}$
Ecosystems	species·yr	$1.79 \cdot 10^{-8}$	$2.79 \cdot 10^{-9}$	$1.40 \cdot 10^{-8}$	$1.03 \cdot 10^{-9}$
Resources	$	$2.56 \cdot 10^{-1}$	$1.04 \cdot 10^{-1}$	$1.42 \cdot 10^{-2}$	$9.52 \cdot 10^{-3}$

C. Heat integration

In this section, we present the hot and cold stream data (**Tables S13 – S15**) and results of the heat integration analysis performed in Aspen Energy Analyzer (**Tables S16 – S18**): area, number of shells, heat load and logarithmic mean temperature difference (LMTD).

Table S13. Hot and cold streams of the base case scenario.

Stream	T_{in} [ºC]	T_{out} [ºC]	Heat load [kJ/h]	Heat transfer coefficient [$kJ \cdot h^{-1} \cdot m^{-2} \cdot {}^{o}C^{-1}$]
1	148.1	40.00	$9.63 \cdot 10^{6}$	453.6
2	127.9	40.00	$7.11 \cdot 10^{6}$	123.7
3	147.0	40.00	$8.89 \cdot 10^{6}$	228.6
4.1	280.3	124.2	$3.30 \cdot 10^{8}$	1168
4.2	124.2	79.59	$1.96 \cdot 10^{8}$	2498
4.3	79.59	35.00	$1.46 \cdot 10^{8}$	2938
5.1	34.88	55.86	$7.78 \cdot 10^{6}$	10227
5.2	55.86	72.27	$6.23 \cdot 10^{6}$	11439
5.3	72.27	80.00	$3.11 \cdot 10^{6}$	12383
6	53.63	228.0	$3.76 \cdot 10^{8}$	1135
7.1	64.37	64.29	$1.59 \cdot 10^{8}$	55164
7.2	64.29	63.87	$4.98 \cdot 10^{5}$	8903
7.3	63.87	63.45	$4.23 \cdot 10^{5}$	8447
8.1	109.06	109.12	$1.44 \cdot 10^{7}$	189346
8.2	109.12	109.17	$1.93 \cdot 10^{7}$	211410
8.3	109.17	109.21	$2.24 \cdot 10^{7}$	227676
8.4	109.21	109.24	$4.12 \cdot 10^{7}$	236253
9	63.45	30.00	$7.94 \cdot 10^{6}$	720.0

Table S14. Hot and cold streams of the Boudouard scenario.

Stream	T_{in} [ºC]	T_{out} [ºC]	Heat load [kJ/h]	Heat transfer coefficient [kJ·h^{-1}·m^{-2}·ºC^{-1}]
1	137.7	40.00	$4.27·10^6$	397.7
2	118.2	40.00	$3.16·10^6$	119.4
3	136.8	40.00	$4.01·10^6$	213.6
4.1	190.3	907.0	$5.18·10^7$	3658
4.2	907.0	1387	$3.77·10^7$	3994
4.3	1387	2000	$5.03·10^7$	3734
5.1	65.41	62.19	$1.29·10^6$	8211
5.2	62.19	52.53	$2.16·10^6$	6794
5.3	52.53	30.00	$4.73·10^6$	6616
6	112.8	228.0	$2.08·10^6$	920.4
7.1	34.92	63.61	$6.45·10^6$	6673
7.2	63.61	75.90	$3.01·10^6$	7341
7.3	75.90	80.00	$2.92·10^6$	10545
8.1	313.9	212.5	$2.09·10^8$	1045
8.2	212.5	76.97	$2.75·10^8$	1119
8.3	76.97	35.00	$1.58·10^8$	2079
9	1999	2000	$1.39·10^8$	720.0
10	108.3	109.3	$1.53·10^8$	720.0
11	66.41	65.41	$1.55·10^8$	720.0

Table S15. Hot and cold streams of the biomass gasification process.

Stream	T_{in} [ºC]	T_{out} [ºC]	Heat load [kJ/h]	Heat transfer coefficient [$kJ\cdot h^{-1}\cdot m^{-2}\cdot {}^{o}C^{-1}$]
1.1	180.7	118.1	$1.83\cdot10^{7}$	1502
1.2	118.1	86.89	$1.02\cdot10^{7}$	1670
1.3	86.89	55.63	$1.27\cdot10^{7}$	2022
1.4	55.63	40.00	$9.63\cdot10^{6}$	2756
2.1	25.00	101.1	$1.69\cdot10^{7}$	19974
2.2	101.1	101.2	1.14E+08	197016
2.3	101.2	333.7	$2.23\cdot10^{7}$	28.28
2.4	333.7	450.0	$1.17\cdot10^{7}$	24.89
3.1	25.00	63.04	$1.62\cdot10^{7}$	19537
3.2	63.04	101.1	$1.63\cdot10^{7}$	21620
3.3	101.1	101.2	2.20E+08	197016
3.4	101.2	250.0	$2.72\cdot10^{7}$	28.07
4	550.0	250.0	$1.04\cdot10^{8}$	110.2
5	700.0	550.0	$5.44\cdot10^{7}$	105.9
6.1	185.7	115.9	$2.62\cdot10^{7}$	888.4
6.2	115.87	60.03	$2.06\cdot10^{7}$	920.7
6.3	60.03	40.00	$9.48\cdot10^{6}$	1079
7.1	185.2	123.4	$2.33\cdot10^{7}$	464.7
7.2	123.4	61.50	$2.28\cdot10^{7}$	477.9
7.3	61.50	40.00	$1.48\cdot10^{7}$	931.7
8.1	250.0	70.81	$9.48\cdot10^{7}$	125.3
8.2	70.81	60.02	$1.03\cdot10^{8}$	5745
8.3	60.02	40.00	$8.74\cdot10^{7}$	9892
9	154.8	40.00	$7.49\cdot10^{6}$	13412
10	156.7	40.00	$8.48\cdot10^{6}$	13412
11	155.3	40.00	$7.70\cdot10^{6}$	13412
12.1	750.1	513.4	$1.99\cdot10^{7}$	83.00
12.2	513.4	276.7	$1.84\cdot10^{7}$	84.36
12.3	276.7	40.00	$1.61\cdot10^{7}$	83.17
13.1	700.0	406.7	$1.42\cdot10^{7}$	15666
13.2	406.7	186.7	$1.22\cdot10^{7}$	2557
13.3	186.7	40.00	$9.48\cdot10^{6}$	30.68

14.1	184.8	61.98	$4.77 \cdot 10^{7}$	247.5
14.2	61.98	53.74	$1.47 \cdot 10^{7}$	1261
14.3	53.74	40.00	$1.71 \cdot 10^{7}$	2033
15.1	25.00	166.7	$6.75 \cdot 10^{6}$	26.18
15.2	166.7	308.3	$7.07 \cdot 10^{6}$	21.66
15.3	308.3	450.0	$7.38 \cdot 10^{6}$	19.08
16	250.0	249.5	$8.59 \cdot 10^{6}$	720.0

Table S16. Heat exchanger network results for the base case scenario (total required heating and cooling are 0 and 104 MW, respectively, for a production of 485 kt/y of methanol).

Unit	$T_{hot,in}$ [ºC]	$T_{hot,out}$ [ºC]	$T_{cold,in}$ [ºC]	$T_{cold,out}$ [ºC]	Area [m^2]	Shells	Heat load [MW]	LMTD [ºC]
1	280.3	112.0	95.2	228.0	25387	55	79.5	31.3
2	280.3	112.0	109.1	109.2	1860	6	27.0	41.5
3	148.1	100.6	24.9	25.0	99	1	1.20	97.5
4	147.0	92.4	24.9	25.0	219	1	1.30	92.1
5	112.0	79.2	55.9	80.0	207	2	2.60	30.4
6	112.0	79.2	55.8	95.2	6840	14	23.6	19.9
7	112.0	79.2	24.2	24.9	336	1	13.8	69.8
8	64.4	63.4	22.1	24.2	361	1	44.5	40.7
9	79.2	76.8	34.9	55.9	108	1	2.20	31.7
10	100.6	65.7	53.6	55.8	393	1	0.90	24.9
11	92.4	73.3	53.6	55.8	308	1	0.40	27.3
12	127.9	40.0	21.9	22.1	1169	3	2.00	49.7
13	63.4	30.0	21.9	22.1	571	2	2.20	20.4
14	76.8	35.0	20.0	21.9	1879	4	38.1	30.8
15	65.7	40.0	20.0	21.9	173	1	0.60	27.4
16	73.3	40.0	20.0	21.9	374	1	0.80	33.3

Table S17. Heat exchanger network results for the Boudouard scenario (total required heating and cooling are 75.9 and 655.5 MW, respectively, for a production of 470 kt/y of methanol).

Unit	$T_{hot,in}$ [ºC]	$T_{hot,out}$ [ºC]	$T_{cold,in}$ [ºC]	$T_{cold,out}$ [ºC]	Area [m^2]	Shells	Heat load [MW]	LMTD [ºC]
1	313.9	132.8	34.9	80.0	91	1	3.4	156
2	313.9	132.8	112.8	228.0	13069	28	66.5	45
3	313.9	132.8	108.5	109.3	3253	8	33.2	85
4	65.4	52.5	20.7	20.7	19	1	1.0	14
5*	3000.0	2999.6	996.0	2000.0	156	1	22.5	1444
6	132.8	115.3	108.3	108.5	5500	11	9.3	38
7	132.8	115.3	20.7	20.7	20	1	0.6	103
8*	1000.0	400.0	190.3	996.0	3568	8	16.3	52
9	115.3	77.0	20.6	20.7	1021	3	21.6	74
10	52.5	30.0	20.6	20.7	58	1	1.3	18
11	77.0	35.0	20.3	20.6	2838	6	43.9	31
12	118.2	40.0	20.0	20.3	545	2	0.9	49
13	137.7	40.0	20.0	20.3	201	1	1.2	55
14	136.8	40.0	20.0	20.3	348	1	1.1	55
15	66.4	65.4	20.0	20.3	4960	10	43.1	46
16*	2999.6	2999.0	1999.0	2000.0	540	1	38.6	1000

*integration takes place completely or partially over 800 ºC so a furnace is considered instead of a regular heat exchanger

Table S18. Heat exchanger network results for the biomass gasification process (total required heating and cooling are 0 and 118 MW, respectively, for a production of 115 kt/y of hydrogen and 52 kt/y of biochar).

Unit	$T_{hot,in}$ [ºC]	$T_{hot,out}$ [ºC]	$T_{cold,in}$ [ºC]	$T_{cold,out}$ [ºC]	Area [m^2]	Shells	Heat load [MW]	LMTD [ºC]
1	61.5	40.0	20.0	23.2	614	1	4.12	28.1
2	113.6	40.0	20.0	23.2	116	1	2.55	46.7
3	180.7	40.0	20.0	23.2	1766	2	2.65	66.6
4	156.8	40.0	23.2	23.8	272	2	2.36	56.1
5	186.7	40.0	23.2	23.8	4823	5	2.63	64.2
6	185.2	178.1	25.0	25.0	38	1	0.75	157
7	700.0	186.7	101.2	250.0	5210	5	7.33	219
8	547.6	280.5	101.1	101.2	2866	3	25.69	293
9	250.0	157.7	101.1	101.2	4081	4	13.54	95.4
10	184.8	156.0	101.1	101.2	663	1	3.11	68.3
11	53.7	40.0	20.0	23.2	393	1	4.74	24.9
12	115.9	40.0	20.0	23.2	321	1	3.66	47.4
13	150.3	115.9	23.8	25.0	144	1	3.59	108
14	62.0	53.7	23.2	23.8	372	1	4.07	34.2
15	155.3	40.0	23.2	23.8	505	2	2.14	55.7
16	700.0	667.6	333.7	450.0	2025	2	3.26	290
17	750.1	579.0	166.7	450.0	2713	3	4.00	353
18	667.6	550.0	101.2	333.7	2913	3	11.86	388
19	185.7	150.3	101.1	101.2	230	1	3.69	65.3
20	113.6	40.0	25.0	101.1	1735	6	6.09	13.7
21	115.9	40.0	25.0	101.1	1799	6	4.69	14.9
22	157.7	111.0	23.8	25.0	1832	2	6.84	108
23	276.7	180.7	101.1	101.1	647	1	1.81	121
24	156.0	62.0	23.8	25.0	2006	2	10.17	75.3
25	178.1	123.4	101.1	101.1	1009	1	5.73	44.1
26	250.0	249.5	101.1	101.1	803	1	23.78	149
27	111.0	40.0	20.0	23.2	3808	4	58.79	45.8
28	280.5	250.0	25.0	101.1	484	1	2.94	201

29	123.4	61.5	23.8	25.0	783	1	6.33	63.3
30	118.1	113.6	23.8	25.0	11	1	0.41	91.5
31	154.8	40.0	23.2	23.8	942	2	2.08	55.5
32	363.8	276.7	25.0	166.7	1582	2	1.88	223
33	579.0	363.8	101.1	101.2	565	1	4.76	360
34	180.7	118.1	101.1	101.1	303	1	5.08	40.5
35	550.0	547.6	101.2	250.0	99	1	0.23	368

D. Economic assessment

In this section, we present the economic data used in the analysis and the capital investment cost methodology employed. Similarly to the environmental assessment, the Cu-ZnO-Al_2O_3 catalyst was not considered in the economic calculations, as its contribution is usually negligible.[16]

D.1. Economic parameters

Table S19. Material and utility prices.

Input	Amount	Units	Reference
H_2 (from wind electricity)	$6.88\cdot10^{0}$	$/kg	[17]
CO_2 (from direct air capture)	$3.62\cdot10^{-1}$	$/kg	[17]
Biomass	$3.00\cdot10^{-2}$	$/dry kg	[18]
O_2 (from air separation)	$1.23\cdot10^{-1}$	$/kg	[15]
Heating (natural gas)	$1.96\cdot10^{0}$	$/GJ	[17]
Cooling (water from 20 to 25 ºC)	$3.60\cdot10^{-1}$	$/GJ	[19]
Electricity (global mix)	$1.04\cdot10^{-1}$	$/kWh	[17]

Table S20. Parameters used for the purchased cost for heat exchangers, furnaces, compressor, distillation columns, trays and vessels.[20]

Process unit	Sizing unit	S_{lower}	S_{upper}	a	b	n
U-tube sheel and tube	Area [m^2]	10	1000	28000	54	1.20
Centrifugal compressor	Power [kW]	75	30000	580000	20000	0.60
Pressure vessel, vertical	Shell mass [kg]	160	250000	11600	34	0.85
Cylindrical furnace	Duty [MW]	0.2	60	130	440	1.80
WGS	3.69	150	250	kg/s	feed	0.67

Table S21. Parameters used for the economic calculations of biomass handling, gasification, PSA, rectisol and WGS units (June 2004 $).[21]

Process unit	C^0 [M$]	S^0	S_{max}	Units	Basis	n
Biomass handling	4.57	17.9	30.6	kg/s	as received biomass	0.77
Gasifier	54.34	17.9	33.3	kg/s	dry biomass	0.77
Rectisol unit	31.59	2.51	8.78	kmol/s	feed	0.63
PSA	7.84	0.29	-	kmol/s	purge gas	0.65
WGS	3.69	150	250	kg/s	feed	0.67
Methanol synthesis	8.09	35.657	-	kg/s	feed	0.65

D.2. Capital investment cost

The fixed capital cost (total fixed capital cost, *CAPEX*) is based on the procedure described in Sinnot and Towler.[20] First, we estimate the purchased equipment costs from the capacity using Eq.(S1):

$$C_{e,i} = a_i + b_i S_i^{n_i} \quad \text{(S1)}$$

Where $C_{e,i}$ is the purchased equipment cost of i in 2010 US \$, a_i, b_i and n_i are constants, and S_i is the size parameter measured in different units depending on the type of equipment i. These data can be found in **Table S20**.

Then, we update these costs from 2010 to 2023 using the Chemical Engineering Plant Cost Index (CEPCI) (Eq.(S2)):

$$\text{Cost in year A} = \text{Cost in year B}\frac{\text{Cost index in year A}}{\text{Cost index in year B}} \quad \text{(S2)}$$

Where Cost in year B and Cost in year A are the purchased equipment costs [\$] for the years 2010 and 2023 and $\text{Cost index in year B}$ (532.9) and $\text{Cost index in year A}$ (797.9) are the CEPCI annual mean values for the corresponding years.

With the updated purchased equipment cost, we add the factors for equipment erection for fluid/fluid-solid processes for (f_{er} = 0.3/0.5), piping (f_p = 0.8/0.6) instrumentation and process control (f_i= 0.3/0.3), electrical work (f_{el} = 0.2/0.2), civil engineering work (f_c = 0.3/0.3), structures and buildings (f_s = 0.2/0.2), lagging, insulation or paint (f_l = 0.1/0.1) and material factor (f_m = 1.3 for stainless steel) to calculate the installed capital cost (C , \$) using Eq.(S3):

$$C = \sum_i C_{e,i} \left[(1+f_p) f_m + f_{er} + f_{el} + f_i + f_c + f_s + f_l \right] \quad \text{(S3)}$$

Then, we add the offsites (*OS* = 0.3/0.4, for a typical large-volume green field chemical plant), design and engineering (*D&E* = 0.3/0.25), and contingency (*X* = 0.1/0.1) and working capital (15% of the fixed capital) to get the *CAPEX* [$] (Eq.(S4)):

$$CAPEX = C(1+OS)(1+D\&E+X)(1+0.15) \quad \text{(S4)}$$

The *CAPEX* of biomass handling, gasification (gasifier), PSA, rectisol unit and WGS reactor is alternatively calculated using **Eq.(S5)** with the parameters in **Table S21**:

$$CAPEX' = \sum_j (1-BOP) C^0 \left(\frac{S_j}{S^0} \right)^{n_j} \quad \text{(S5)}$$

Where $CAPEX'$ is the cost (M$), BOP is the balance of plant cost (0.2), that includes site conditioning, utility infrastructure, etc., C^0 is the base unit cost (**Table S21**), S^0 is the base capacity (**Table S21**) and n_j is the scaling factor.

The CAPEX of the RB reactor is estimated from a blast furnace using economy of scale (**Eq.(S6)**):

$$CAPEX'' = C^{bf} \left(\frac{S^{rb}}{S^{bf}} \right)^{n^{bf}} \quad \text{(S6)}$$

Where $CAPEX''$ is the cost (M$), C^{bf} is the base cost of the blast furnace (346 M$), S^{bf} is the base volume of the blast furnace (3700 m^3), S^{rb} is the RB reactor volume (337 m^3) and n^{bf} is the is the scaling factor (0.9).

We calculate the annualization factor (or annual capital charge ratio, *ACCR*) considering a 30-year lifetime of the plant and an interest rate of 0.1 as described in **Eq.(S7)**:

$$ACCR = \frac{\left[i(1+i)^n\right]}{\left[(i+1)^n - 1\right]} \quad \text{(S7)}$$

Finally, the product of the *ACCR* (0.1061) and the *CAPEX* results in the annualized capital investment (*ACC*, \$/y) (**Eq.(S8)**):

$$ACC = (CAPEX + CAPEX')\cdot ACCR \quad \text{(S8)}$$

The *OPEX* was computed from the unitary methanol raw material and energy consumption (F_i) and their associated costs ($price_i$) found in Tables S1, S2, S3 and S14 and using **Eq.(S18)**. In addition, the total fixed production costs ($FOPEX$), were computed using **Eqs.(S9)-(S17)** as described in Sinnot and Towler and considering 50000 \$/operator, 3 shifts and 4.8 operators/shift:

$$labor\ costs = operating\ labor + supervision + direct\ overhead \quad \text{(S9)}$$

$$operating\ labor = salary \cdot operator \cdot shift \quad \text{(S10)}$$

$$supervision = operating\ labor \cdot 0.25 \quad \text{(S11)}$$

$$direct\ overhead = (operating\ labor + supervision) \cdot 0.40 \quad \text{(S12)}$$

$$maintenance = C \cdot 0.03 \quad \text{(S13)}$$

$$plant\ overhead = (labor\ costs + maintenance) \cdot 0.65 \quad \text{(S14)}$$

$$tax\ \&\ insurance = C(1+OS) \cdot 0.02 \quad \text{(S15)}$$

$$interest = (CAPEX \cdot 0.15) \cdot 0.06 \quad \text{(S16)}$$

$$FOPEX = labor\ costs + maintenance + plant\ overhead + tax\ \&\ insurance + interest \quad \text{(S17)}$$

The OPEX is then computed using Eq.(S17):

$$OPEX = \sum_i F_i \cdot price_i + FOPEX \tag{S18}$$

Finally, the total annualized cost (TAC) is calculated as the sum of the $OPEX$ and the $ACCR$ (**Eq.(S19)**):

$$TAC = OPEX + ACCR \tag{S19}$$

E. Economic results

In this section, we show the economic contributions of each process unit, raw material and energy.

Table S22. Individual purchased cost results of the base case green methanol process (2010 dollars).

Unit	Sizing	Units	Amount	Purchased cost [M$]
Heat exchanger (U-tube shell and tube)	462	m^2	55	6.22
Heat exchanger (U-tube shell and tube)	310	m^2	6	0.48
Heat exchanger (U-tube shell and tube)	99.0	m^2	1	0.04
Heat exchanger (U-tube shell and tube)	219	m^2	1	0.06
Heat exchanger (U-tube shell and tube)	103	m^2	2	0.08
Heat exchanger (U-tube shell and tube)	489	m^2	14	1.67
Heat exchanger (U-tube shell and tube)	336	m^2	1	0.09
Heat exchanger (U-tube shell and tube)	361	m^2	1	0.09
Heat exchanger (U-tube shell and tube)	108	m^2	1	0.04
Heat exchanger (U-tube shell and tube)	393	m^2	1	0.10
Heat exchanger (U-tube shell and tube)	308	m^2	1	0.08
Heat exchanger (U-tube shell and tube)	390	m^2	3	0.29
Heat exchanger (U-tube shell and tube)	285	m^2	2	0.15
Heat exchanger (U-tube shell and tube)	470	m^2	4	0.46
Heat exchanger (U-tube shell and tube)	173	m^2	1	0.05
Heat exchanger (U-tube shell and tube)	374	m^2	1	0.09
Compressor (centrifugal)	2260	kW	1	2.64
Compressor (centrifugal)	2351	kW	1	2.69
Compressor (centrifugal)	2288	kW	1	2.65
Compressor (centrifugal)	1322	kW	1	2.07
Compressor (centrifugal)	3022	kW	1	3.03
Compressor (centrifugal)	6898	kW	1	4.60
Compressor (centrifugal)	2260	kW	1	2.64
Compressor (centrifugal)	2351	kW	1	2.69
Distillation column (pressure vessel)	17528	kg	1	0.15
Tray (sieve)	3.91	m	52	0.27
Furnace (cyclindrical)	9.31	MW	1	0.73
Flash unit (pressure vessel)	100560	kg	1	0.62

Flash unit (pressure vessel)	950	kg	1	0.02

Table S23. Total CAPEX contributions of the base case green methanol process updated to 2023.

Unit	Purchase cost [M$]	CAPEX [M$]
Heat exchanger (U-tube shell and tube)	15.0	117
Compressor (centrifugal)	26.5	207
Distillation column (pressure vessel)	0.63	4.95
Furnace (cyclindrical)	1.09	8.55
Flash unit (pressure vessel)	0.96	7.53
Methanol reactor	-	44.8

Table S24. Individual purchased cost results of the RB integrated with green methanol process (2010 dollars).

Unit	Sizing	Units	Amount	Purchased cost [M$]
Heat exchanger (U-tube shell and tube)	91	m^2	1	0.04
Heat exchanger (U-tube shell and tube)	13069	m^2	28	3.20
Heat exchanger (U-tube shell and tube)	3253	m^2	8	0.81
Heat exchanger (U-tube shell and tube)	19	m^2	1	0.03
Furnace (cyclindrical)	22.5	MW	1	6.26
Heat exchanger (U-tube shell and tube)	5500	m^2	11	1.34
Heat exchanger (U-tube shell and tube)	20	m^2	1	0.03
Heat exchanger (U-tube shell and tube)	3568	m^2	8	0.88
Heat exchanger (U-tube shell and tube)	1021	m^2	3	0.26
Heat exchanger (U-tube shell and tube)	58	m^2	1	0.04
Heat exchanger (U-tube shell and tube)	2838	m^2	6	0.69
Heat exchanger (U-tube shell and tube)	545	m^2	2	0.15
Heat exchanger (U-tube shell and tube)	201	m^2	1	0.06
Heat exchanger (U-tube shell and tube)	348	m^2	1	0.09
Heat exchanger (U-tube shell and tube)	4960	m^2	10	1.21
Furnace (cyclindrical)	38.6	MW	1	16.8
Compressor (centrifugal)	1020	kW	1	1.86
Compressor (centrifugal)	1061	kW	1	1.89

Compressor (centrifugal)	1036	kW	1	1.87
Compressor (centrifugal)	970	kW	1	1.82
Compressor (centrifugal)	2012	kW	1	2.50
Compressor (centrifugal)	7542	kW	1	4.82
Distillation column (pressure vessel)	16134	kg	1	0.14
Tray (sieve)	3.63	m	52	0.24
Furnace (cyclindrical)	12	MW	1	0.87
Furnace (cyclindrical)	41	MW	1	2.23
Flash unit (pressure vessel)	100560	kg	1	0.62
Flash unit (pressure vessel)	950	kg	1	0.02

Table S25. Total CAPEX contributions of the RB integrated with green methanol synthesis process updated to 2023.

Unit	Purchase cost [M$]	CAPEX [M$]
Heat exchanger (U-tube shell and tube)	13.2	105
Compressor (centrifugal)	22.1	177
Distillation column (pressure vessel)	0.57	4.55
Furnace (cyclindrical)	34.5	276
Flash unit (pressure vessel)	0.96	7.69
Methanol reactor	-	43.0
RB reactor (blast furnace)	-	61.3

Table S26. Individual purchased cost results of the biomass gasification process (2010 dollars).

Unit	Sizing	Units	Amount	Purchased cost [M$]
Heat exchanger (U-tube shell and tube)	614	m^2	1	0.15
Heat exchanger (U-tube shell and tube)	116	m^2	1	0.04
Heat exchanger (U-tube shell and tube)	1766	m^2	2	0.43
Heat exchanger (U-tube shell and tube)	272	m^2	2	0.10
Heat exchanger (U-tube shell and tube)	4823	m^2	5	1.17
Heat exchanger (U-tube shell and tube)	38	m^2	1	0.03
Heat exchanger (U-tube shell and tube)	5210	m^2	5	1.27
Heat exchanger (U-tube shell and tube)	2866	m^2	3	0.69
Heat exchanger (U-tube shell and tube)	4081	m^2	4	0.99
Heat exchanger (U-tube shell and tube)	663	m^2	1	0.16
Heat exchanger (U-tube shell and tube)	393	m^2	1	0.10
Heat exchanger (U-tube shell and tube)	321	m^2	1	0.08
Heat exchanger (U-tube shell and tube)	144	m^2	1	0.05
Heat exchanger (U-tube shell and tube)	372	m^2	1	0.09
Heat exchanger (U-tube shell and tube)	505	m^2	2	0.14
Heat exchanger (U-tube shell and tube)	2025	m^2	2	0.49
Heat exchanger (U-tube shell and tube)	2713	m^2	3	0.66
Heat exchanger (U-tube shell and tube)	2913	m^2	3	0.71
Heat exchanger (U-tube shell and tube)	230	m^2	1	0.06
Heat exchanger (U-tube shell and tube)	1735	m^2	6	0.46
Heat exchanger (U-tube shell and tube)	1799	m^2	6	0.47
Heat exchanger (U-tube shell and tube)	1832	m^2	2	0.44
Heat exchanger (U-tube shell and tube)	647	m^2	1	0.16
Heat exchanger (U-tube shell and tube)	2006	m^2	2	0.49
Heat exchanger (U-tube shell and tube)	1009	m^2	1	0.25
Heat exchanger (U-tube shell and tube)	803	m^2	1	0.19
Heat exchanger (U-tube shell and tube)	3808	m^2	4	0.92
Heat exchanger (U-tube shell and tube)	484	m^2	1	0.12
Heat exchanger (U-tube shell and tube)	783	m^2	1	0.19
Heat exchanger (U-tube shell and tube)	11	m^2	1	0.03
Heat exchanger (U-tube shell and tube)	942	m^2	2	0.23
Heat exchanger (U-tube shell and tube)	1582	m^2	2	0.38

Heat exchanger (U-tube shell and tube)	565	m^2	1	0.14
Heat exchanger (U-tube shell and tube)	303	m^2	1	0.08
Heat exchanger (U-tube shell and tube)	99	m^2	1	0.04
Compressor (centrifugal)	12	MW	1	7.12
Compressor (centrifugal)	41	MW	1	6.94
Compressor (centrifugal)	15507	MW	1	6.88
Compressor (centrifugal)	14816	MW	1	4.75
Compressor (centrifugal)	14591	MW	1	2.52
Compressor (centrifugal)	7323	MW	1	2.51
Compressor (centrifugal)	2044	MW	1	2.47
Compressor (centrifugal)	2023	MW	1	7.12
Compressor (centrifugal)	1959	MW	1	6.94

Table S27. Total CAPEX contributions of the biomass gasification process updated to 2023.

Unit	Purchase cost [M$]	CAPEX [M$]
Heat exchanger (U-tube shell and tube)	18.0	143
Compressor (centrifugal)	26.5	177
Biomass handling	-	13.1
Gasifier	-	146
WGS reactor	-	3.62
Rectisol unit	-	57.3
PSA	-	30.8

Table S28. OPEX contributions of the base case green methanol process.

Contribution	Amount	Units	Cost [M$/y]
H_2	94.2	t/y	648
CO_2	704	t/y	255
Heating	0.00	GJ/h	0.00
Cooling	376	GJ/h	1.02
Electricity	18.1	MW	15.1
Fixed OPEX	-	-	16.9

Table S29. OPEX contributions of the RB integrated with green methanol synthesis process.

Contribution	Amount	Units	Cost [M$/y]
H_2	62.7	t/y	432
CO_2	352	t/y	127
Heating	270	GJ/h	2.7
Cooling	347	GJ/h	0.83
Electricity	13.6	MW	11.4
Fixed OPEX	-	-	26.5

Table S30. OPEX contributions of the biomass gasification process.

Contribution	Amount	Units	Cost [M$/y]
Biomass	200	t/y	6.00
O_2	352	t/y	8.74
Heating	0.00	GJ/h	0.00
Cooling	81.1	GJ/h	0.22
Electricity	11.1	MW	9.26
Fixed OPEX	-	-	51.7

F. Reverse Boudouard reactor volume

We estimate the reaction temperature and reactor volume from the kinetic data from Dai et al. using Eqs.(S20)-(S21) for a fixed CO_2 conversion of 89%:[3]

$$V = \frac{-Q_v}{k_{VRM}} \ln(1 - X) \quad \text{(S20)}$$

$$k_{VRM} = A \cdot \exp\left(-\frac{E_a}{RT}\right) \quad \text{(S21)}$$

Where V is the reactor volume (m^3), Q_v is the total volumetric flow rate at 55 bar (m^3/min), X is the fractional conversion of CO_2 (0.89), k_{VRM} is the kinetic constant (min^{-1}), A is the preexponential factor (1088.6 min^{-1}), E_a is the activation energy (148900 J/mol), R is the gas constant (8.314 J/mol/K) and T is the reaction temperature (K).

The results are shown in **Figure S4**. The volume of the reactor sharply decreases with temperatures higher than 1100 ºC (36000 m^3), reaching minimum values around 2000 ºC (337 m^3). However, since a higher temperature also implies an increased energy consumption, we also analyzed the effect on the cost of the methanol (**Figure S5**).

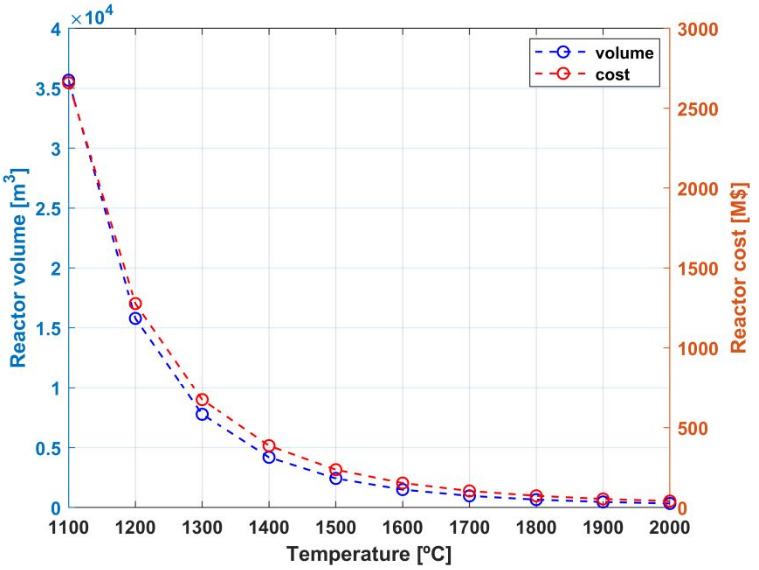

Figure S4. Relationship between reaction temperature, reactor volume and cost for the reverse Boudouard reaction with an 89% conversion and a reaction pressure of 55 bar.

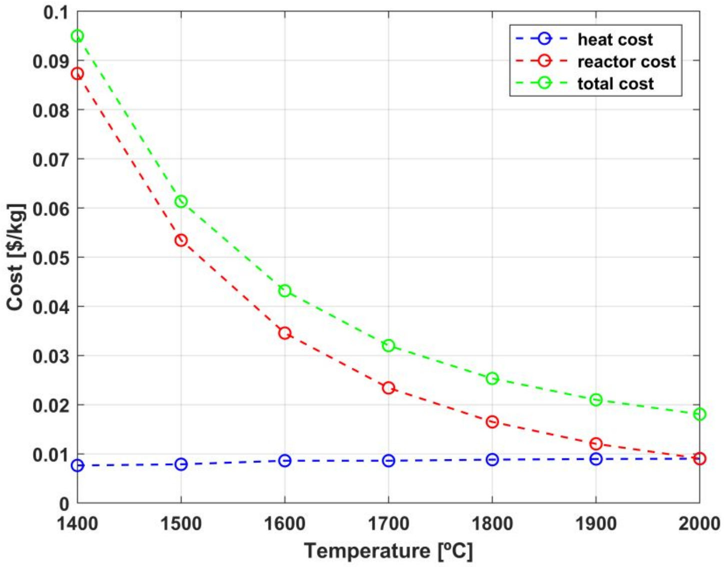

Figure S5. Relationship between reaction temperature, reactor CAPEX and heating cost for the reverse Boudouard reaction with an 89% conversion and a reaction pressure of 55 bar.

Here we can observe that while the heating cost contribution stays around 0.01 $/kg of methanol independently of the reaction temperature, the reactor cost drastically decreases, down to around 0.01 $/kg of methanol at 2000 ºC. Hence, for our RB reactor design we choose a temperature of 2000 ºC which yields a reactor volume of 337 m^3.

G. Reverse Boudouard reactor temperature effect

In this section, we show the effect of the RB reactor temperature on the economic and climate change impact results of producing green methanol using the equilibrium model from Hunt et al.[22]

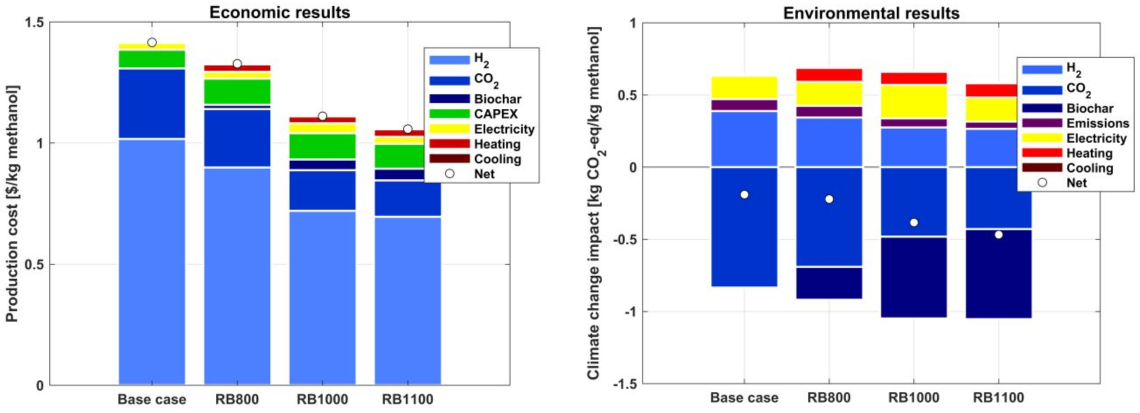

Figure S6. RB temperature effect on the carbon footprint (right) and cost (left) of green methanol production. The base case scenario represents standard unintegrated green methanol synthesis from DAC CO_2 and wind-powered electrolytic H_2. The RB800, RB1000 and RB1100 scenarios consider integration of green methanol with the RB reaction and are associated with different temperatures in the said unit (800, 1000 and 1100 ºC, respectively).

Higher temperatures result in higher conversions in the RB reactor which in turn reduce the amount of electrolytic H_2 required in the methanol synthesis step. Since this H_2 is the main driver of cost in green methanol synthesis, the cost of the overall synthesis sharply decreases. Other effects in this improvement are the reduction of expensive DAC CO_2 in favor of cheap biochar, a higher CAPEX compared with the unintegrated base case and slightly higher cost due to the additional heating utilities and compression.

In terms of carbon footprint, a similar behavior is shown, where less H_2 and CO_2 are consumed while higher heating, electricity and biochar usage are required. The main driver here is the low carbon footprint of biochar (assuming mass allocation from the biogas gasification process),

which makes de overall climate change impact also decrease as the temperature, and hence, conversion, in the RB increases.

References

(1) González-Garay, A.; Frei, M. S.; Al-Qahtani, A.; Mondelli, C.; Guillén-Gosálbez, G.; Pérez-Ramírez, J. Plant-to-Planet Analysis of CO2-Based Methanol Processes. Energy Environ. Sci. 2019, 12 (12), 3425–3436, DOI 10.1039/c9ee01673b.

(2) Bussche, K. M. V.; Froment, G. F. A Steady-State Kinetic Model for Methanol Synthesis and the Water Gas Shift Reaction on a Commercial Cu/ZnO/Al2O3Catalyst. J. Catal. 1996, 161 (1), 1–10, DOI 10.1006/jcat.1996.0156.

(3) Dai, H.; Zhao, H.; Chen, S.; Jiang, B. A Microwave-Assisted Boudouard Reaction: A Highly Effective Reduction of the Greenhouse Gas Co2 to Useful Co Feedstock with Semi-Coke. Molecules 2021, 26 (6), DOI 10.3390/molecules26061507.

(4) Doherty, W.; Reynolds, A.; Kennedy, D. Aspen plus Simulation of Biomass Gasification in a Steam Blown Dual Fluidised Bed. Mater. Process Energy 2013, 212–220.

(5) Mutlu, Ö. Ç.; Zeng, T. Challenges and Opportunities of Modeling Biomass Gasification in Aspen Plus: A Review. Chem. Eng. Technol. 2020, 43 (9), 1674–1689, DOI 10.1002/ceat.202000068.

(6) Vassilev, S. V.; Baxter, D.; Andersen, L. K.; Vassileva, C. G. An Overview of the Chemical Composition of Biomass. Fuel 2010, 89 (5), 913–933, DOI 10.1016/j.fuel.2009.10.022.

(7) Atikah, M. S. N.; Taufiq Yap, Y. H.; Ilyas, R. A.; Harun, R. Optimization of Algae Residues Gasification: Experimental and Theoretical Approaches. J. Phys. Conf. Ser. 2022, 2259 (1), 012012, DOI 10.1088/1742-6596/2259/1/012012.

(8) Schmid, J. C.; Benedikt, F.; Fuchs, J.; Mauerhofer, A. M.; Müller, S.; Hofbauer, H. Syngas for Biorefineries from Thermochemical Gasification of Lignocellulosic Fuels and

Residues—5 Years' Experience with an Advanced Dual Fluidized Bed Gasifier Design. Biomass Convers. Biorefinery 2021, 11 (6), 2405–2442, DOI 10.1007/s13399-019-00486-2.

(9) Rahim, D. A.; Fang, W.; Wibowo, H.; Hantoko, D.; Susanto, H.; Yoshikawa, K.; Zhong, Y.; Yan, M. Review of High Temperature H2S Removal from Syngas: Perspectives on Downstream Process Integration. Chem. Eng. Process. - Process Intensif. 2023, 183 (November 2022), 109258, DOI 10.1016/j.cep.2022.109258.

(10) Ersöz, A.; DurakÇetin, Y.; Sarıoğlan, A.; Turan, A. Z.; Mert, M. S.; Yüksel, F.; Figen, H. E.; Güldal, N.; Karaismailoğlu, M.; Baykara, S. Z. Investigation of a Novel & Integrated Simulation Model for Hydrogen Production from Lignocellulosic Biomass. Int. J. Hydrogen Energy 2018, 43 (2), 1081–1093, DOI 10.1016/j.ijhydene.2017.11.017.

(11) Medrano-García, J. D.; Ruiz-Femenia, R.; Caballero, J. A. Optimal Carbon Dioxide and Hydrogen Utilization in Carbon Monoxide Production. J. CO2 Util. 2019, 34, 215–230, DOI 10.1016/j.jcou.2019.05.005.

(12) Weidema, B. P. Comparing Three Life Cycle Impact Assessment Methods from an Endpoint Perspective. J. Ind. Ecol. 2015, 19 (1), 20–26, DOI 10.1111/jiec.12162.

(13) Medrano-García, J. D.; Charalambous, M. A.; Guillén-Gosálbez, G. Economic and Environmental Barriers of CO2-Based Fischer-Tropsch Electro-Diesel. ACS Sustain. Chem. Eng. 2022, 10 (36), 11751–11759, DOI 10.1021/acssuschemeng.2c01983.

(14) Keith, D. W.; Holmes, G.; St. Angelo, D.; Heidel, K. A Process for Capturing CO2 from the Atmosphere. Joule 2018, 2 (8), 1573–1594, DOI 10.1016/j.joule.2018.05.006.

(15) Medrano-García, J. D.; Giulimondi, V.; Ceruti, A.; Zichittella, G.; Pérez-Ramírez, J.; Guillén-Gosálbez, G. Economic and Environmental Competitiveness of Ethane-Based Technologies for Vinyl Chloride Synthesis. ACS Sustain. Chem. Eng. 2023, 11 (35), 13062–13069, DOI 10.1021/acssuschemeng.3c03006.

(16) Pérez-Fortes, M.; Schöneberger, J. C.; Boulamanti, A.; Tzimas, E. Methanol Synthesis Using Captured CO2as Raw Material: Techno-Economic and Environmental Assessment. Appl. Energy 2016, 161, 718–732, DOI 10.1016/j.apenergy.2015.07.067.

(17) Nabera, A.; José Martín, A.; Istrate, R.; Pérez-Ramírez, J.; Guillén-Gosálbez, G. Integrating Climate Policies in the Sustainability Analysis of Green Chemicals. Green Chem. 2024, 26 (11), 6461–6469, DOI 10.1039/d4gc00392f.

(18) Spath, P.; Aden, A.; Eggeman, T.; Ringer, M.; Wallace, B.; Jechura, J. Biomass to Hydrogen Production Detailed Design and Economics Utilizing the Battele Columbus Laboratory Indirectly-Heated Gasifier; 2005. https://www.nrel.gov/docs/fy05osti/37408.pdf.

(19) Richard A., T.; Shaeiwitz, J. A.; Bhattacharyya, D.; Wallace B., W. 8.3 Utility Costs. In Analysis, Synthesis and Design of Chemical Processes; Prentice Hall, 2018.

(20) Towler, G.; Sinnott, R. K. Chemical Engineering Design; Elsevier Ltd, 2013, DOI 10.1016/C2009-0-61216-2.

(21) Onel, O.; Niziolek, A. M.; Elia, J. A.; Baliban, R. C.; Floudas, C. A. Biomass and Natural Gas to Liquid Transportation Fuels and Olefins (BGTL+C2-C4): Process Synthesis and Global Optimization. Ind. Eng. Chem. Res. 2015, 54 (1), 359–385, DOI 10.1021/ie503979b.

(22) Hunt, J.; Ferrari, A.; Lita, A.; Crosswhite, M.; Ashley, B.; Stiegman, A. E. Microwave-Specific Enhancement of the Carbon−Carbon Dioxide (Boudouard) Reaction. J. Phys. Chem. 2013, 117 (51), 26871–26880, DOI https://doi.org/10.1021/jp4076965.
